# Supplementary material for: Patient experience of NHS health checks: a systematic review and qualitative synthesis
Source: BMJ Open. 2017 Aug 11;7(8):e017169. doi: 10.1136/bmjopen-2017-017169 (PMC5724113; doi:10.1136/bmjopen-2017-017169)
Supplement: Supplementary file 2 [file bmjopen-2017-017169supp002.pdf]

Appendix 1 – Search strategies

Database	Search strategy
Ovid Medline	1. health check*.tw.<br>2. (diabetes adj3 screen*).tw.<br>3. (cardiovascular adj3 screen*).tw.<br>4. (population adj2 screen*).tw.<br>5. (risk factor adj3 screen*).tw.<br>6. (opportunistic adj3 screen*).tw.<br>7. medical check*.tw.<br>8. general check*.tw.<br>9. periodic health exam*.tw.<br>10. annual exam*.tw.<br>11. annual review*.tw.<br>12. NHSHC.tw.<br>13. 1 or 2 or 3 or 4 or 5 or 6 or 7 or 8 or 9 or 10 or 11 or 12<br>14. cardiovascular adj3 prevention.tw.<br>15. (primary care or general practice or primary healthcare).tw<br>16. 14 and 15<br>17. Cardiovascular Diseases/ AND Primary Prevention/<br>18. 16 or 17<br>19. 13 or 18
PubMed	1. health check*<br>2. diabetes screen*<br>3. cardiovascular screen*<br>4. population screen*<br>5. risk factor screen*<br>6. opportunistic screen*<br>7. medical check*<br>8. general check*<br>9. periodic health exam*<br>10. annual exam*<br>11. annual review*<br>12. NHSHC<br>13. 1 or 2 or 3 or 4 or 5 or 6 or 7 or 8 or 9 or 10 or 11 or 12<br>14. Cardiovascular Diseases AND Primary Prevention[MeSH Terms]<br>15. "primary care"[Text Word] OR "general practice"[Text Word] OR "primary healthcare"[Text Word])<br>16. (cardiovascular[Text Word] AND prevention[Text Word])<br>17. #15 and #16<br>18. #14 or #17<br>19. #13 or #18
Ovid Embase	1. health check*.tw.<br>2. (diabetes adj3 screen*).tw.<br>3. (cardiovascular adj3 screen*).tw.<br>4. (population adj2 screen*).tw.

	5. (risk factor adj3 screen*).tw.<br>6. (opportunistic adj3 screen*).tw.<br>7. medical check*.tw.<br>8. general check*.tw.<br>9. periodic health exam*.tw.<br>10. annual exam*.tw.<br>11. annual review*.tw.<br>12. NHSHC.tw.<br>13. periodic medical examination/<br>14. 1 or 2 or 3 or 4 or 5 or 6 or 7 or 8 or 9 or 10 or 11 or 12 or 13<br>15. cardiovascular adj3 prevention.tw.<br>16. (primary care or general practice or primary healthcare).tw<br>17. 15 and 16<br>18. cardiovascular disease/ AND primary prevention/<br>19. 17 or 18<br>20. 14 or 19
Ovid HMIC	1 "health check*".af.<br>2 health checks/<br>3 (cardiovascular or vascular or heart or diabetes or stroke).af.<br>4 (screen* or risk).af.<br>5 3 AND 4<br>6 1 OR 2 or 5<br>7 cardiovascular adj3 prevention.tw.<br>8 (primary care or general practice or primary healthcare).tw<br>9 7 and 8<br>10 Cardiovascular diseases/ AND exp preventive medicine/<br>11 9 or 10<br>12 6 or 11
EBSCO CINAHL	S10 S1 OR S2 OR S9<br>S9 S5 OR S8<br>S8 S6 AND S7<br>S7 (MH "Preventive Health Care+")<br>S6 (MH "Cardiovascular Diseases+")<br>S5 S3 AND S4<br>S4 "primary care" or "general practice" or "primary healthcare"<br>S3 TX cardiovascular N3 prevention<br>S2 (diabetes N3 screen*) OR (cardiovascular N3 screen*) OR (population N2 screen*) OR (risk factor N3 screen*) OR (opportunistic N3 screen*) OR “medical check*” OR “general check*” OR “periodic health exam*” OR “annual exam*” OR "annual review*" OR NHSHC<br>S1 health check*
EBSCO Global Health	S10 S6 OR S19 OR S3 Limiters - Publication Year: 2016<br>S9 S7 AND S8<br>S8 DE "preventive medicine"<br>S7 DE "cardiovascular diseases"<br>S6 S4 AND S5<br>S5 "primary care" or "general practice" or "primary healthcare"

	S4 TX cardiovascular N3 prevention<br>S3 S1 OR S2<br>S2 (diabetes N3 screen*) OR (cardiovascular N3 screen*) OR (population N2 screen*) OR (risk factor N3 screen*) OR (opportunistic N3 screen*) OR “medical check*” OR “general check*” OR “periodic health exam*” OR “annual exam*” OR "annual review*" OR NHSHC<br>S1 health check*
HDAS PsycInfo	1 "health check*".af.<br>2 PHYSICAL EXAMINATION/<br>3 HEALTH SCREENING/<br>4 "diabetes screen*".af<br>5 "cardiovascular screen*".af<br>6 "population screen*".af<br>7 ("opportunistic* screen*" OR "risk factor screen*").af<br>8 ("medical check*" OR "general check*" OR "periodic health exam*" OR "annual exam*" OR "annual review*" OR NHSHC).af<br>9 1 OR 2 OR 3 OR 4 OR 5 OR 6 OR 7 OR 8<br>10 cardiovascular.ti,ab<br>11 prevention.ti,ab<br>12 10 AND 11<br>13 CARDIOVASCULAR DISORDERS/<br>14 PREVENTIVE MEDICINE/<br>15 13 AND 14<br>16 12 OR 15<br>17 9 OR 16
Web of Science, Science Citation Index	“health check*” OR “diabetes screen*” OR “cardiovascular screen*” OR “population screen*” OR “risk factor screen*” OR “Opportunistic screen*” OR “medical check*” OR “general check*” OR “periodic health exam*” OR “annual exam*” OR “annual review*” OR NHSHC<br>OR<br>(Cardiovascular NEAR/3 prevention) AND (“primary care” OR “general practice” OR “primary healthcare”)<br>Limit to: England, Scotland, Wales, North Ireland
Cochrane Library (Wiley)	#1 "health check*"<br>#2 (diabetes next/3 screen*) or (cardiovascular next/3 screen*) or (population next/2 screen*) or (opportunistic next/2 screen*) or ("risk factor" next/3 screen*) or "medical check*" or "general check*" or "periodic health exam*" or "annual exam*" or "annual review*" or NHSHC<br>#3 cardiovascular adj3 prevention.tw.<br>#4 (primary care or general practice or primary healthcare).tw<br>#5 #3 and #4<br>#6 MeSH descriptor: [Cardiovascular Diseases] this term only<br>#7 MeSH descriptor: [Primary Prevention] explode all trees<br>#8 #6 and #7<br>#9 #5 or #8<br>#10 #1 or #2 or #9

NHS Evidence	“health check*” OR cardiovascular prevention primary care
TRIP database	“health check*” OR cardiovascular prevention primary care
Google Scholar	"nhs health check"<br>cardiovascular “health check”<br>cardiovascular prevention “primary care”
Google	"nhs health check"<br>cardiovascular prevention “primary care”<br>cardiovascular “health check”
Clinical trials.gov and ISRCDN registry	“health check”
