# Supplementary material for: Patient experience of NHS health checks: a systematic review and qualitative synthesis
Source: BMJ Open. 2017 Aug 11;7(8):e017169. doi: 10.1136/bmjopen-2017-017169 (PMC5724113; doi:10.1136/bmjopen-2017-017169)
Supplement: Supplementary file 3 [file bmjopen-2017-017169supp003.pdf]

Appendix 2 – Quality assessment of quantitative studies

Author, date	Study addressed a clearly focused issue	Use of an appropriate method	Recruitment	Exposure measurement	Outcome measurement	Confounding factors	Applicability to England	Overall
Baker 2014	⬤	●	⬤	●	●	•	●	High
Corlett 2015	⬤	⬤	⬤	⬤	⬤	•	•	High
Cowper 2013	⬤	•	•	•	•	•	●	Low
Krska 2015	⬤	⬤	⬤	●	⬤	•	●	High
LGA – East Riding 2015	⬤	•	•	•	•	•	•	Low
NHS Greenwich	●	●	●	●	●	•	•	Medium
'A picture of Health'	●	•	•	•	•	•	•	Low
Taylor 2012	⬤	⬤	●	⬤	⬤	•	●	High
Trivedy 2016	●	●	●	⬤	●	•	•	Medium

• Low ● Medium ⬤ High
