# Supplementary material for: Patient experience of NHS health checks: a systematic review and qualitative synthesis
Source: BMJ Open. 2017 Aug 11;7(8):e017169. doi: 10.1136/bmjopen-2017-017169 (PMC5724113; doi:10.1136/bmjopen-2017-017169)
Supplement: Supplementary file 1 [file bmjopen-2017-017169supp001.pdf]

Appendix 3 – Quality assessment of qualitative studies

Author and date	Study addressed a clearly focused issue	Appropriateness of qualitative method	Design	Recruitment	Consideration of relationship between research and participants	Ethical issues	Rigor of data analysis	Clarity of statement of findings	Overall
Alford 2010	⬤	⬤	⬤	•	•	•	•	●	Medium
Baker 2014	⬤	⬤	⬤	⬤	n/a	•	⬤	⬤	High
Chipchase 2011	⬤	⬤	⬤	●	●	⬤	⬤	●	High
Corlett 2015	⬤	⬤	⬤	●	•	●	●	●	Medium
Greenwich 2011	⬤	⬤	⬤	●	•	•	•	●	Medium
Ismail and Atkin 2015	⬤	⬤	⬤	⬤	●	⬤	⬤	⬤	High
Jenkinson 2015	⬤	⬤	⬤	⬤	•	⬤	⬤	⬤	High
Krska 2015	⬤	⬤	●	⬤	n/a	●	●	●	Medium
McNaughton 2015	⬤	⬤	⬤	⬤	•	⬤	⬤	⬤	High
Oswald 2010	⬤	⬤	⬤	●	•	●	●	●	Medium
Perry 2014	⬤	⬤	⬤	⬤	•	•	⬤	⬤	High
Riley 2015 (JPH)	⬤	⬤	⬤	●	●	⬤	⬤	⬤	High
Riley 2015 (BMC HSR)	⬤	⬤	⬤	●	●	⬤	⬤	⬤	High
Shaw 2015	⬤	⬤	⬤	●	•	⬤	⬤	⬤	High
Strutt 2011	⬤	⬤	⬤	⬤	⬤	⬤	⬤	⬤	High

• Low ● Medium ⬤ High
