# Supplementary material for: Patient experience of NHS health checks: a systematic review and qualitative synthesis
Source: BMJ Open. 2017 Aug 11;7(8):e017169. doi: 10.1136/bmjopen-2017-017169 (PMC5724113; doi:10.1136/bmjopen-2017-017169)
Supplement: Author's manuscript [file bmjopen-2017-017169.draft_revisions.pdf]

BMJ Open is committed to open peer review. As part of this commitment we make the peer review history of every article we publish publicly available.

When an article is published we post the peer reviewers' comments and the authors' responses online. We also post the versions of the paper that were used during peer review. These are the versions that the peer review comments apply to.

The versions of the paper that follow are the versions that were submitted during the peer review process. They are not the versions of record or the final published versions. They should not be cited or distributed as the published version of this manuscript.

BMJ Open is an open access journal and the full, final, typeset and author-corrected version of record of the manuscript is available on our site with no access controls, subscription charges or pay-per-view fees (http://bmjopen.bmj.com).

If you have any questions on BMJ Open's open peer review process please email editorial.bmjopen@bmj.com

BMJ Open

Patient experience of NHS Health Checks: a systematic review and qualitative synthesis

Journal:	*BMJ Open*
Manuscript ID	bmjopen-2017-017169
Article Type:	Research
Date Submitted by the Author:	05-Apr-2017
Complete List of Authors:	Usher-Smith, Juliet; University of Cambridge, The Primary Care Unit<br>Harte, Emma; RAND Europe<br>MacLure, Calum; RAND Europe<br>Martin, Adam; Academic Unit of Health Economics, Leeds Institute of Health Sciences<br>Saunders, Katie; University of Cambridge,<br>Meads, Catherine; Anglia Ruskin University, Faculty of Health, Social Care and Education<br>Walter, Fiona; University of Cambridge, Dept of Public Health and Primary Care<br>Griffin, Simon; University of Cambridge, The Primary Care Unit; University of Cambridge, MRC Epidemiology Unit<br>Mant, Jonathan; University of Cambridge, The Primary Care Unit
**Primary Subject Heading**:	Public health
Secondary Subject Heading:	Cardiovascular medicine, Communication, Qualitative research
Keywords:	NHS Health Check, patient experience, systematic review, qualitative synthesis

SCHOLARONE™ Manuscripts

Patient experience of NHS Health Checks: a systematic review and qualitative synthesis

Usher-Smith JA[1], Harte E[2], MacLure C[2], Martin A[3], Saunders CL[1], Meads C[4], Walter FM[1], Griffin SJ[1,5], Mant J[1].

[1]The Primary Care Unit, Institute of Public Health, University of Cambridge, Box 113 Cambridge Biomedical Campus, Cambridge, CB2 0SR, UK

[2]RAND Europe, Westbrook Centre, Milton Road, Cambridge, CB4 1YG, UK

[3]Academic Unit of Health Economics, Leeds Institute of Health Sciences, University of Leeds, Leeds, LS2 9LJ, UK

[4]Faculty of Health, Social Care and Education, Anglia Ruskin University

[5]MRC Epidemiology Unit, University of Cambridge, Institute of Metabolic Science, Cambridge, CB2 0QQ, UK

Correspondence to: Juliet Usher-Smith jau20@medschl.cam.ac.uk

Key words: NHS Health Check, patient experience, systematic review, qualitative synthesis

For peer review only

ABSTRACT (300 words)

Objective: To review the experiences of patients attending NHS Health Checks in England.

Design: A systematic review of quantitative and qualitative studies with a narrative synthesis of quantitative studies and thematic synthesis of qualitative studies.

Data sources: An electronic literature search of Medline, Embase, Health Management Information Consortium (HMIC), Cumulative Index of Nursing and Allied Health Literature (CINAHL), Global Health, PsycInfo, Web of Science, OpenGrey, the Cochrane Library, NHS Evidence, Google Scholar, Google, Clinical Trials.gov and the ISRCTN registry to 09/11/16 with no language restriction and manual screening of reference lists of all included papers.

Inclusion criteria: Primary research reporting experiences of patients who have attended NHS Health Checks.

Results: 20 studies met the inclusion criteria, 9 reporting quantitative data and 15 qualitative data. There were consistently high levels of reported satisfaction in surveys, with over 80% feeling that they had benefited from the process. Data from qualitative studies showed that the NHS Health Check had been perceived to act as a wake-up call for many who reported having gone on to make substantial lifestyle changes which they attributed to the NHS Health Check. However, some had been left with a feeling of unmet expectations, were confused about or unable to remember their risk scores, found the lifestyle advice too simplistic and non-personalised, or were confused about follow-up.

Conclusions: Whilst participants were generally very supportive of the NHS Health Check programme and examples of behaviour change were reported, there are a number of areas where improvements could be made. These include greater clarity around the aims of the programme within the promotional material, more pro-active support for lifestyle change, and greater appreciation of the challenges of communicating risk and the limitations of relying on the risk score alone as a trigger for facilitating behaviour change.

For peer review only - http://bmjopen.bmj.com/site/about/guidelines.xhtml

Strengths and limitations of this study

- This is the first study to systematically review quantitative and qualitative studies that consider the experiences of patients who have attended NHS Health Checks.
- The use of broad inclusion criteria and the systematic search of multiple databases and the grey literature allowed us to include studies that had not been published in peer reviewed scientific journals.
- The included studies were of varying quality.
- The quantitative studies reporting responses to surveys had response rates between 23% and 43%, making them at risk of responder bias.
- The qualitative studies included small, selected groups of participants whose expressed views were likely to be affected by both recall bias and social desirability bias.

peer review only

For peer review only - http://bmjopen.bmj.com/site/about/guidelines.xhtml

INTRODUCTION

The NHS Health Check programme is one of the largest current prevention initiatives in England. Introduced in 2009 to improve cardiovascular disease (CVD) risk factors through behavioural change and treatment informed by risk stratification, it became a mandated public health service in 2013. Local authorities are now responsible for offering an NHS Health Check to individuals aged 40-74 without existing cardiovascular disease, diabetes or hypertension every five years. The NHS Health Check itself consists of three components: risk assessment, communication of risk and risk management. Risk tools are used to establish the individual's risk of developing CVD and diabetes. That assessment is then used to raise awareness of relevant risk factors and inform discussion about the lifestyle and medical approaches best suited to managing the individual's risk of disease. Based on modelling studies of cross-sectional data it was estimated that the programme could prevent 1,600 heart attacks and strokes, at least 650 premature deaths, and over 4,000 new cases of diabetes each year with an estimated cost per quality adjusted life year (QALY) of approximately £3,000[1]. However, whether NHS Health Checks represent an efficient use of scarce health promotion resources has been questioned.

Alongside clinical effectiveness and safety, patient experience is increasingly recognised worldwide as one of the three elements of high-quality healthcare[2–4]. As well as enabling a better understanding of current problems with healthcare delivery, informing continuous improvement and redesign of services and helping professionals reflect on practice, a recent systematic review has shown that patient experience is positively associated with self-rated and objectively measured health outcomes; adherence to recommended medication and treatments; preventative care; healthcare resource use; technical quality-of-care delivery; and adverse events[5,6]. There is also an association at the organizational level; general practices that provide higher quality clinical care (measured through higher QOF performance) are also those in which reported patient experience is better. Understanding patients' experiences of NHS Health Checks is, therefore, central to understanding the implementation of the programme, its potential impact over the first eight years, and ways in which it might be improved.

Since the introduction of the NHS Health Check programme, a growing number of both quantitative and qualitative studies reporting patients' experiences of NHS Health Checks have been published. This article provides the first systematic synthesis of these studies.

For peer review only - http://bmjopen.bmj.com/site/about/guidelines.xhtml

METHODS

We performed a systematic literature review following a study protocol (available on request) that followed the PRISMA guidelines.

Search strategy

We used the results of an existing literature review conducted by Public Health England covering the period from 1st January 1996 to 9th November 2016 supplemented by a search of the Web of Science, Science Citation Index and OpenGrey covering the same period, and hand searching of the reference lists of all included publications. The PHE literature review included the following sources: Medline, Embase, Health Management Information Consortium (HMIC), Cumulative Index of Nursing and Allied Health Literature (CINAHL), Global Health, PsycInfo, the Cochrane Library, NHS Evidence, Google Scholar, Google, Clinical Trials.gov and the ISRCTN registry. Full details of all the search strategies are shown in Appendix 1. No language restrictions were applied.

Study selection

To be included studies had to be primary research reporting the opinions or experiences of people who had attended NHS Health Checks. Commentaries, editorials and opinion pieces were excluded.

The selection of studies was performed in a two-stage process. First, the titles and abstracts were screened to identify studies relevant to the NHS Health Check. This stage had already been completed by a senior information scientist at PHE for those identified in the literature review conducted by PHE. One reviewer (EH) followed this process for the additional citations identified from the Web of Science database.

In the second stage two researchers (JUS and AM) reviewed the full texts of all studies identified as relevant to the NHS Health Checks to select those reporting the opinions or experiences of people who had attended NHS Health Checks. Where it was unclear whether or not these inclusion criteria were met for any given study, we discussed those studies at consensus meetings with the wider research team.

Data extraction, quality assessment and synthesis

For peer review only - http://bmjopen.bmj.com/site/about/guidelines.xhtml

Data on the study design, time period, recruitment methods, participants, analysis, and quantitative results were extracted independently for each study by two reviewers (JUS + EH/CMa) onto data extraction forms developed to minimise bias. The quality of all included studies was assessed at the same time using the Critical Appraisal Skills Programmes (CASP) checklist for qualitative research[7] or a checklist combining the CASP checklists for cohort studies and randomised-controlled trials for the quantitative studies. For studies that included both quantitative and qualitative methods, quality assessment was completed separately for both aspects of the study.

We synthesised the qualitative data using thematic synthesis. Following reading and re-reading of the included studies, this synthesis included three stages[8]: 1) coding of the findings of the primary studies; 2) organisation of these codes into related areas to develop descriptive themes; and 3) the development of analytical themes. The initial coding of the findings of the primary studies was performed by at least two researchers (JUS + EH/CMa). These findings were then discussed with members of the wider research team and the subsequent stages were an iterative process with both the descriptive and analytical themes developed through a series of meetings involving researchers from a range of clinical and non-clinical backgrounds. To allow an appreciation of the primary data, we have included illustrative quotations from the original studies alongside the analytical themes in this report.

RESULTS

From an initial 18,524 titles and abstracts, 178 articles were identified as potentially relevant to the NHS Health Checks and were reviewed at full text level (Figure 1). Of those, we excluded 162. The most common reasons for excluding papers were that they did not include any relevant data, were duplicates or commentaries, or did not describe NHS Health Checks. Four additional articles were identified through citation searching. This review is, therefore, based on 20 articles.

Quantitative results from patient satisfaction questionnaires

Of those 20 articles, nine include quantitative results from surveys of participants who had attended NHS Health Checks[9–17]. The details of these nine articles are shown in Table 1 and full details of the quality assessment in Appendix 2. Four are high quality journal articles published in peer reviewed journals in which questionnaires were sent to all those who had attended an NHS Health Check in either general practices[9,10] or pharmacies[16,17]. Response

For peer review only - http://bmjopen.bmj.com/site/about/guidelines.xhtml

rates were between 23.4% and 43%. A fifth study of the views of those attending outreach clinic at cricket groups was also published in a peer reviewed journal but does not report the methods in detail or the response rate[12]. Another is a report of a service evaluation in which the views of ethnic minority participants were particularly sought[13] and the final three are low quality case study reports which have not reported methods or response rate[11,14,15].

All included studies report high levels of satisfaction, with almost all respondents across the range of settings reporting a positive experience and over 80% feeling that they had benefited from the process. When reported, nine out of ten respondents also felt that they had been given enough time, had been able to ask all their questions, and would recommend the process to others.

Qualitative data on patient experience

Patient experience was also reported in 15 qualitative studies. Three performed content analysis on free-text responses provided in surveys[9,10,13] whilst the others conducted focus groups or interviews with between 8 and 45 participants. Ten are journal articles published in peer reviewed journals[9,10,17–24] four are research reports of service evaluations[13,25–27], and one is a Masters thesis[28]. All recruited people who had attended NHS Health Checks either through invitations sent out from general practices or from community settings. Most included approximately equal numbers of men and women. Three studies had particularly sought to describe the experiences of those from ethnic minority groups[13,23,24]. Further details of the design and methods used in those studies are given in Table 2 and the full quality assessment in Appendix 3.

Thematic synthesis of these 15 studies identified five main analytical themes: 1) The NHS Health Check as a potential trigger for behaviour change; 2) Unmet expectations; 3) Limited understanding of the risk score; 4) Preference for better information; and 5) Confusion around follow-up. The primary articles contributing to each of those themes are shown in Table 3 and details of each of the themes given below.

1. The NHS Health Check as a potential trigger for behaviour change

Participants variously described the NHS Health Check as a "*wake-up call*"[13,21], a "*reality check*"[21], a "*kind of turning point*"[28], a *"nudge"*[17], an "*eye-opener*"[24], or a "*prompt*"[26] which helped bring patients' health into focus by highlighting underlying health issues of which

For peer review only - http://bmjopen.bmj.com/site/about/guidelines.xhtml

they were not necessarily cognizant[10] and making them aware that there were lifestyle-related diseases to which they may be susceptible and which they may be able to prevent[24].

> "*It's really good. It makes you aware of what problems are around. What you can get and that. It is really good. It teaches you..it's an eye-opener for people who would want to do things properly*" [24]

For some, this reputedly led on to behaviour change, with many of the studies citing examples of participants who had reported making changes that they attributed to having attended the NHS Health Check[18,21–25,29]. These included changes to diet, cutting down on smoking, decreasing alcohol intake and increasing physical activity.

> *"I've changed my diet um and, and lost a stone in weight I think as a result actually. So I'm quite happy with that, that makes me feel even healthier"* [22]

> "*Having the results of the check, I've actually started to go to [swimming baths] a couple of times, so I've made some progress….and I've actually felt better in meself.*" [21]

In general, dietary changes were perceived to be the easiest changes to make, particularly small changes that did not cause too much disruption to their daily routines[29] and there was recognition that changing behaviour was hard, with a number of barriers identified (Box 1).

2. Unmet expectations

Despite this potential influence on behaviour, a strong theme throughout many of the studies was that of unmet expectations that some participants were left with at the end of the NHS Health Check.

For many, this arose from confusion about the purpose of the NHS Health Check. The comparison made between the NHS Health Check and an 'MOT' in the promotional material and the use of the term 'Health Check' left many expecting the NHS Health Check to include a more general wide-ranging assessment of health and not just risk of cardiovascular disease[10,18,26].

For peer review only - http://bmjopen.bmj.com/site/about/guidelines.xhtml

"*I just assumed that they would test you for everything when you were there. My perception of reading through things was that it was going to be a good overhaul, you know overall body check for everything.*" [26]

"*As a general health check it was not a series of tests as I expected. Only centred around the result of a blood test. Not comprehensive as I would have expected*" [9]

Additional specific areas that participants had been expecting or thought should be covered included: a Well woman check[26]; diabetes checks for all[23,26,27]; cancer screening[18,26,28]; an assessment of mental well-being[18]; an ECG[18]; testing for anaemia[18]; discussion around health conditions that impacted on their daily lives, such as joint and back pain[28]; and chronic long-term conditions[27].

3. Limited understanding of the risk score

Whilst some participants reported improved understanding of CVD risk following an NHS Health Check[9,17,24], a common theme throughout the studies was participants' limited understanding of the risk score.

Across many of the studies there was evidence that a large number of participants were either not able to recall being provided with a risk estimate at all[9,18,19], found the risk score confusing[10,22,26], or had interpreted it incorrectly[18,21,22,28].

"*my cholesterol is high…and, I had a score saying sixteen per cent diabetes in ten years. What does that mean? I've got no idea what that means. It sounds bad because it's higher than it's meant to be but is it?*"[22]

"'*My QRisk score is 11 per cent. But after getting someone to Google it for me, we still have no idea what it means. It should be explained better in a letter from the Doctor*" [10]

The score itself also appeared to have little meaning or significance for most participants. Low scores (<20% 10-year estimated risk) were sometimes perceived as meaning there was nothing to worry about[21], but participants with low-risk scores were as likely to report being worried or anxious after receiving the scores as those with high-risk scores[22]. When

For peer review only - http://bmjopen.bmj.com/site/about/guidelines.xhtml

For peer review only

describing their motivation to change behaviour, in general participants also described how it was not necessarily related to their risk score, and how even a high risk score was not necessarily enough to motivate them to try and change[21,28].

> "*Sometimes you need a reason and I think it was like me, I needed a reason [to change] and isn't it sad that showing me the percentage wasn't reason enough for me to give up [smoking].*" [21]

4. Preferences for better information

Most participants reported receiving lifestyle advice within the NHS Health Check. Many, however, felt it was too simple, brief, superficial or generic and felt that they would have benefitted from more detail and more personalised information[13,17,21,22,24,28,29].

> *"And it was that kind of information which was the kind of the bit beyond, you know, eat less, exercise more, don't smoke, don't drink .. .that would have been useful ..the kind of advice that was on offer was actually very, um, simple"* [22]

For some this lack of personalised information led to confusion and uncertainty[28], with some feeling that they had received mixed messages about their health[18] and been left unsure about what actions they should take[25]. This was not a universal view, however, with some seeing the value in being provided with 'common knowledge' again as it afforded a fresh way of looking at their lifestyle and, in one study the simplicity of the information appeared to encourage participants to make changes to their behaviour[21].

> *"So I thought it was very helpful it was very informative and it was thought-provoking, it just gave us some fresh view on things, because you can get very easily into doing what you think is okay"* [28]

In most cases the lifestyle advice had been provided face-to-face but participants also valued, or felt it would have been helpful to have received, written information both for their own reference and also as a means to encourage behaviour change among friends and family[10,21,26].

For peer review only - http://bmjopen.bmj.com/site/about/guidelines.xhtml

"Well I suppose it's good to have a question and answer thing cos you can have somebody explain it to you. But I suppose you could, something written'd be quite useful." [18]

5. Confusion around follow-up

The final theme related to confusion over follow-up[13,24,25]. This was particularly seen amongst participants who had attended NHS Health Checks in community settings. Individuals felt unsure about what steps should be taken next, specifically in relation to whether they needed to contact their GP or if their GP would contact them if any causes for concern had been identified[25]. Participants also reported a lack of sufficient information on follow-up and sign-posting to other NHS services[13].

"She never said go and see your doctor. She ran out of some leaflets and she circled them and said go on the Internet" [13]

Some participants also reported that they would have liked their healthcare professionals to be more pro-active in supporting them to make lifestyle changes and felt there should have been on-going follow-up and monitoring[18,24,27].

DISCUSSION

Principal findings

This study is the first systematic review of patient experience of NHS Health Checks. It shows that, amongst those who respond to patient satisfaction surveys, there are consistently very high levels of satisfaction with NHS Health Checks reported, with over 80% feeling that they had benefited from the process. However, despite these overall high levels of satisfaction, there was evidence from interviews that some participants were left with a feeling of unmet expectations. For some this appeared to arise from confusion about the purpose of the NHS Health Check whilst others had been expecting a more general assessment of health. The cardiovascular risk score also appeared to generate confusion: it was poorly understood, interpreted differently among individuals with the same level of risk, and seemed to have little meaning or significance for people in terms of how to use it to think about their health and future planning. Most participants reported receiving lifestyle information within the NHS Health Check but for many it was regarded as too simple and not sufficiently personalised. Nevertheless, there was evidence that the NHS Health Check was

For peer review only - http://bmjopen.bmj.com/site/about/guidelines.xhtml

perceived to act as a wake-up call for many participants who had gone on to make substantial lifestyle changes which they attributed to the NHS Health Check.

Strengths and Limitations

The main strengths of this review are the systematic search of multiple electronic databases, the manual searching of the reference lists of all included studies, and the thematic approach to synthesis of the qualitative data. By including searches of the grey literature and the internet alongside electronic repositories we were able to include studies that have not been published in peer reviewed scientific journals, reducing the risk of selective reporting bias. Whilst we cannot exclude the possibility that additional local evaluations may have been performed or that we overlooked studies at the screening stage, we think it unlikely that they would substantially alter the main findings. Choosing to conduct a thematic synthesis for the qualitative research also ensured that we used a systematic approach to identify common themes across the studies and interpret those findings. Although some argue against the synthesis of qualitative research on the grounds that the findings of individual studies are de-contextualised and the concepts identified in one setting are not applicable to another[30], this approach enabled us not only to provide a synthesis of the evidence to inform practice but also to develop additional interpretations and conceptual insights beyond the findings of the primary studies.

The main limitations relate to the included studies. The quantitative surveys were of varying quality with response rates only reported in four of the nine studies and comparison between responders and non-responders only in one. Where reported, the response rates varied between 23% and 43%. Although survey response rates alone are a poor indicator of bias[31,32], the included studies are, therefore, all at risk of responder bias, and may represent the views of those more engaged with preventive healthcare or with particularly strong opinions. Many also measured patient satisfaction rather than patient experience. Unlike patient experience data which aim to avoid value judgments, patient satisfaction is a broad and often ill-defined concept that is multi-dimensional and influenced by a range of factors, including cultural norms, health status and prior experience of health care[33]. Reports of patient satisfaction can, therefore, vary widely between different patients in identical situations and in one study in which patients were asked a single question about how satisfied overall they were with their primary care practice, only 4.6% of the variance in their satisfaction ratings was a result of

For peer review only - http://bmjopen.bmj.com/site/about/guidelines.xhtml

differences between practices[34]. Unlike patient experience data, improvements in patient satisfaction data are also not associated with improvements in care quality[35].

The qualitative studies also included small, selected groups of participants whose expressed views are likely to be affected by both recall bias (systematic errors due to inaccuracy of recollections about NHS Health Checks) and social desirability bias (the tendency of interviewees to give responses that they think might be viewed favourably by the interviewer). By virtue of the fact that they have chosen to take part in medical research the participants may also be more interested in their health than the general population so their views may not reflect the full range of views and experiences of those attending NHS Health Checks.

Comparison with existing literature

The high levels of satisfaction reported are consistent with those reported for other NHS services, for example the General Practice Patient Survey in which the median overall satisfaction score was 86.2[6] The discrepancy between the very high levels of reported satisfaction in surveys and the more negative comments made in face-to-face interviews is also consistent with previous research in other areas of health care. For example, studies have found that positive survey responses can mask important negative dimensions which patients subsequently express qualitatively[36–38] and that patients often give positive satisfaction ratings even in the context of a negative experience where they believe the poor care is not under the direct control of the person they are evaluating[39]. The interpretation of 'good' absolute patient feedback scores should, therefore, not lead to complacency and the conclusion that improvements need not be considered.

The challenges of communicating risk are also well known[40] and the finding that knowing the CVD risk score was not sufficient to motivate behaviour change is consistent with guidance from the National Institute for Health and Clinical Excellence (NICE) on behaviour change[41] and previous systematic reviews[42,43].

Implications for clinicians, policymakers and future research

Whilst participants were generally very supportive of the NHS Health Check programme and examples of behaviour change were reported, this study highlights a number of areas where improvements could be made. In particular, the finding that a number of patients were

For peer review only - http://bmjopen.bmj.com/site/about/guidelines.xhtml

unaware of the programme or had misunderstood the extent and purpose of the NHS Health Check suggests that more proactive communications may be needed to raise awareness of the programme overall and that patients need additional clarity about the programme when being invited. The expectation that the NHS Health Check would be a more general health check also raises questions about whether the programme should be expanded to cover other areas of health. The lack of clarity around follow-up and reports that participants would have liked their healthcare professionals to be more pro-active in supporting them to make lifestyle changes additionally suggests that there are potential missed opportunities to support behaviour change. This may be in part due to a lack of appropriate services to refer patients to but may also reflect a need for additional training for those delivering the NHS Health Check. Finally, the confusion around the CVD risk score amongst patients highlights the potential limitations of relying on the risk score alone as a trigger for facilitating behaviour change within NHS Health Checks and the need for adequate training and time for healthcare professionals to help patients understand their risk in line with best practice guidance[44,45]. Further research is also needed to determine whether different communication strategies, such as heart age[46], improve understanding and subsequent behaviour change.

ACKNOWLEDGEMENTS

We thank our patient and public representatives Kathryn Lawrence and Chris Robertson for providing helpful comments on the findings and the NHS Health Checks Expert Scientific and Clinical Advisory Panel working group for providing us with the initial literature search conducted by Public Health England. We would also like to thank Anna Knack, Research Assistant at RAND Europe, for her excellent research support, and Emma Pitchforth for her helpful comments on our analysis.

Contributors

JUS developed the protocol, screened articles for inclusion, extracted and synthesised the quantitative and qualitative data, interpreted the findings and wrote the first draft of the manuscript. EH screened articles for inclusion, extracted and synthesised the qualitative data, interpreted the findings and critically revised the manuscript. CMa extracted and synthesised the qualitative data and critically revised the manuscript. AM screened articles for inclusion, interpreted the findings and critically revised the manuscript. CS, CM, FW, SG and JM developed the protocol, interpreted the findings and critically revised the manuscript.

For peer review only - http://bmjopen.bmj.com/site/about/guidelines.xhtml

Funding

This work was funded by a grant from Public Health England. JUS was funded by an NIHR Clinical Lectureship and FW by an NIHR Clinician Scientist award. The views expressed in this publication are those of the authors and not necessarily those of the NHS, the NIHR or the Department of Health.

All researchers were independent of the funding body and the funder had no role in data collection, analysis and interpretation of data; in the writing of the report; or decision to submit the article for publication.

Data sharing

All data are available from the reports or authors of the primary research. No additional data is available.

Competing Interests

All authors have completed the Unified Competing Interest form at *www.icmje.org/coi_disclosure.pdf* (available on request from the corresponding author) and declare that (1) they have no support from or relationships with companies that might have an interest in the submitted work in the previous 3 years; (2) their spouses, partners, or children have no financial relationships that may be relevant to the submitted work; and (3) they have no non-financial interests that may be relevant to the submitted work.

The corresponding author has the right to grant on behalf of all authors and does grant on behalf of all authors, an exclusive licence on a worldwide basis to the BMJ Publishing Group Ltd and its Licensees to permit this article (if accepted) to be published in BMJ editions and any other BMJPGL products and sub-licences to exploit all subsidiary rights, as set out in our licence (http://resources.bmj.com/bmj/authors/checklists-forms/licence-for-publication).

All authors had full access to all of the data in the study and can take responsibility for the integrity of the data and the accuracy of the data analysis

The corresponding author affirms that the manuscript is an honest, accurate, and transparent account of the study being reported; that no important aspects of the study have been omitted;

For peer review only - http://bmjopen.bmj.com/site/about/guidelines.xhtml

and that any discrepancies from the study as planned (and, if relevant, registered) have been explained.

FIGURE LEGENDS

Figure 1. PRISMA flow diagram

REFERENCES

1 Department of Health. Economic Modelling for Vascular Checks. 2008.www.healthcheck.nhs.uk/document.php?o=225

2 Professor the Lord Darzi. High quality care for all: NHS Next Stage Review final report. 2008. http://webarchive.nationalarchives.gov.uk/20130107105354/http:/www.dh.gov.uk/en/Publicationsandstatistics/Publications/PublicationsPolicyAndGuidance/DH_085825

3 Institute of Medicine. Corssing the quality chasm: a new health system for the 21st century. 2001. https://www.nationalacademies.org/hmd/~/media/Files/Report Files/2001/Crossing-the-Quality-Chasm/Quality Chasm 2001 report brief.pdf

4 Campbell SM, Roland MO, Buetow SA. Defining quality of care. *Soc Sci Med* 2000;**51**:1611–25.

5 Doyle C, Lennox L, Bell D. A systematic review of evidence on the links between patient experience and clinical safety and effectiveness. *BMJ Open* 2013;**3**:1–18.

6 Llanwarne NR, Abel GA, Elliott MN, *et al.* Relationship between clinical quality and patient experience: analysis of data from the english quality and outcomes framework and the National GP Patient Survey. *Ann Fam Med* 2013;**11**:467–72.

7 Critical Appraisal Skills Programme (CASP) Checklists. http://www.casp-uk.net/casp-tools-checklists

8 Thomas J, Harden A. Methods for the thematic synthesis of qualitative research in systematic reviews. *BMC Med Res Methodol* 2008;**8**:45.

9 Krska J, du Plessis R, Chellaswamy H. Views and experiences of the NHS Health Check provided by general medical practices: cross-sectional survey in high-risk patients. *J Public Health (Bangkok)* 2015;**37**:210–7.

10 Baker C, Loughren EA, Crone D, *et al.* Patients' perceptions of a NHS Health Check in the primary care setting. *Qual Prim Care* 2014;**22**:232–7.

11 Cowper. The NHS Health Check Leadership Forum : Summary and Findings. *NHS Heal Check Leadersh Forum* 2013.

For peer review only - http://bmjopen.bmj.com/site/about/guidelines.xhtml

12 Trivedy C, Vlaev I, Seymour R, *et al.* An evaluation of opportunistic health checks at cricket matches: the Boundaries for Life initiative. *Sport Soc* 2016;**437**:1–9.

13 NHS Greenwich. Evaluation of NHS Health Check PLUS Community Outreach Programme in Greenwich. 2011;:1–61.

14 LGA - East Riding. Public health transformation twenty months on: adding value to tackle local health needs. Published Online First: 2015.http://www.local.gov.uk/documents/10180/6869714/L15_15+Public+health+transformation+twenty+months+on_WEB_39693.pdf/7bb8060e-9a7b-4b85-8099-e854be74cfb5

15 Health Diagnostics. A Picture Of Health NHS Health Checks Case Study : Case study Introduction : National inequalities in health check provision. 2014.

16 Taylor J, Krska J, MacKridge A. A community pharmacy-based cardiovascular screening service: Views of service users and the public. *Int J Pharm Pract* 2012;**20**:277–84.

17 Corlett SA, Krska J. Evaluation of NHS Health Checks provided by community pharmacies. *J Public Health (Bangkok)* 2015;:1–8.

18 Ismail H, Atkin K. The NHS Health Check programme: Insights from a qualitative study of patients. *Heal Expect* 2015;**19**:345–55.

19 Jenkinson CE, Asprey A, Clark CE, *et al.* Patients' willingness to attend the NHS cardiovascular health checks in primary care: a qualitative interview study. *BMC Fam Pract* 2015;**16**:33.

20 McNaughton RJ, Oswald NT, Shucksmith JS, *et al.* Making a success of providing NHS Health Checks in community pharmacies across the Tees Valley: a qualitative study. *BMC Health Serv Res* 2011;**11**:1–7.

21 Perry C, Thurston M, Alford S, *et al.* The NHS health check programme in England: a qualitative study. *Health Promot Int* 2014;**31**:106–15.

22 Riley R, Coghill N, Montgomery A, *et al.* Experiences of patients and healthcare professionals of NHS cardiovascular health checks: A qualitative study. *J Public Health (Bangkok)* 2015;**38**:543–51.

23 Riley R, Coghill N, Montgomery A, *et al.* The provision of NHS health checks in a community setting: an ethnographic account. *BMC Health Serv Res* 2015;**15**:1–10.

24 Shaw RL, Pattison HM, Holland C, *et al.* Be SMART: examining the experience of implementing the NHS Health Check in UK primary care. *BMC Fam Pract* 2015;**16**:1.

25 Alford S, Catherine P. Knowsley at Heart community NHS health checks : Behaviour

For peer review only - http://bmjopen.bmj.com/site/about/guidelines.xhtml

change evaluation. 2010.http://chesterrep.openrepository.com/cdr/handle/10034/118071

26 Chipchase L, Hill P, Waterall J. An insight into the NHS Health Check Programme in Birmingham; Summar report. 2011.

27 Oswald N, Mcnaughton R, Watson P, *et al.* Tees Vascular Assessment Programme Evaluation. 2010.

28 Strutt E. *Patient-centred care: patients' experiences of and responses to the National Health Service (NHS) Health Check programme in general practice.* 2011.http://etheses.dur.ac.uk/3246

29 McNaughton RJ, Shucksmith J. Reasons for (non)compliance with intervention following identification of 'high-risk' status in the NHS Health Check programme. *J Public Heal (United Kingdom)* 2015;**37**:218–25.

30 Britten N, Campbell R, Pope C, *et al.* Using meta ethnography to synthesise qualitative research: a worked example. *J Health Serv Res Policy* 2002;**7**:209–15.

31 Groves RM, Peytcheva E. The impact of nonresponse rates on nonresponse bias: A meta-analysis. *Public Opin Q* 2008;**72**:167–89.

32 Davern M. Nonresponse rates are a problematic indicator of nonresponse bias in survey research. *Health Serv Res* 2013;**48**:905–12.

33 Cleary P. Satisfaction may not suffice!: a commentary on a patient's perspective. *Int J Tech Assess Heal Care* 1998;**14**:35–7.

34 Salisbury C, Wallace M, Montgomery AA. Patients' experience and satisfaction in primary care: secondary analysis using multilevel modelling. *BMJ* 2010;**341**:c5004–c5004.

35 Sitzia J, Wood N. Patient satisfaction: a review of issues and concepts. *Soc Sci Med* 1997;**45**:1829–43.

36 Roland M, Campbell J, Burt J. Improving patient experience in primary care: a multi-method programme of research on the measurement and improvement of patient experience. *NIHR PGfAR*;:In press.

37 Dougall A, Russell A, Rubin G, *et al.* Rethinking patient satisfaction: patient experiences of an open access flexible sigmoidoscopy service. *Soc Sci Med* 2000;**50**:53–62.

38 Burt J, Newbould J, Abel G, *et al.* Investigating the meaning of 'good' or 'very good' patient evaluations of care in English general practice: a mixed methods study. *BMJ Open* 2017;:In press.

For peer review only - http://bmjopen.bmj.com/site/about/guidelines.xhtml

39 Schneider H, Palmer N. Getting to the truth? Researching user views of primary health care. *Health Policy Plan* 2002;**17**:32–41.

40 Waldron C-A, van der Weijden T, Ludt S, *et al.* What are effective strategies to communicate cardiovascular risk information to patients? A systematic review. *Patient Educ Couns* 2011;**82**:169–81.

41 National Institute for Health and Care Excellence. Public Health Guideline - Behaviour change: individual approaches. 2014. www.nice.org.uk/guidance/ph49

42 Willis A, Davies M, Yates T, *et al.* Primary prevention of cardiovascular disease using validated risk scores: a systematic review. *J R Soc Med* 2012;**105**:348–56.

43 Usher-Smith JA, Silarova B, Schuit E, *et al.* Impact of provision of cardiovascular disease risk estimates to healthcare professionals and patients: a systematic review. *BMJ Open* 2015;**5**:e008717.

44 Zipkin DA, Umscheid CA, Keating NL, *et al.* Evidence-based risk communication: a systematic review. *Ann Intern Med* 2014;**161**:270–80.

45 Fagerlin A, Zikmund-Fisher BJ, Ubel PA. Helping patients decide: Ten steps to better risk communication. *J Natl Cancer Inst* 2011;**103**:1436–43.

46 Wells S, Kerr A, Eadie S, *et al.* 'Your Heart Forecast': a new approach for describing and communicating cardiovascular risk? *Heart* 2010;**96**:708–13.

For peer review only

For peer review only - http://bmjopen.bmj.com/site/about/guidelines.xhtml

Table 1. Features of and findings from studies reporting results of participant satisfaction questionnaires

Study/ year	Type of article	Setting	*n*	Recruitment	Response rate (%)	Participant satisfaction	Overall quality
Baker 2014[10]	Journal article	83 general practices	1,011	Survey sent to all patients who had completed an NHS Health Check within a 2-month period	43	91.7% rated the overall experience highly<br>90.2% felt it was worth attending	High
Corlett 2015[17]	Journal article	Pharmacy-based NHS Health Checks	190	Survey sent to all those who had attended an NHS Health Check within a 4 week period	35	Almost all viewed their experience positively<br>92% felt they were given enough time<br>94% were able to ask all their questions<br>3% had unanswered questions<br>99% understood everything	High
Cowper 2013[11]	Case study	NHS Health Checks in County Durham	483	No details provided	Not given	82.2% were very satisfied<br>99.6% would recommend to others	Low
Krska 2015[9]	Journal article	16 general practices in North West England	434	All patients with estimated 10-year CVD risk > 20% from the 16 practices were sent a postal survey regardless of whether they had attended an NHS Health Check or not	23.4	85.6% felt they had benefited<br>89.6% felt they were given enough time<br>90.2% were able to ask all their questions<br>93.6% felt comfortable discussing their lifestyle<br>91.9% understood everything discussed<br>13.5% would have liked more support changing lifestyle<br>7.4% had concerns that had not been dealt with	High
LGA – East Riding[14]	Case study	Outreach NHS Health Check clinics at leisure centres, community centres and workplace settings	Not given	No details provided	Not given	92% rated experience as good or very good	Low
NHS Greenwich[13]	Evaluation report	Outreach clinics	540	Questionnaire distributed at community NHS Health Check venues	Not given	97% satisfied or very satisfied overall<br>90% likely or very likely to return if invited back	Medium
NHS Greenwich[13]	Evaluation report	Outreach clinics	72	Questionnaire distributed at community NHS Health Check venues	Not given	95% satisfied or very satisfied	Medium
'A picture of Health'[15]	Case study	General practice-based pilot of point-of-care NHS Health Checks in Tyne and Wear	281	No details provided	Not given	'High levels' of satisfaction<br>78% likely to recommend to others	Low
Taylor 2012[16]	Journal article	Pharmacy-based NHS Health Checks	97	Pharmacists gave invitation packs to all those who attended an NHS Health Check during the first six months	37.4	Almost all reported a positive experience<br>99% felt they had benefited<br>99.7% felt they were given enough time<br>99% felt comfortable discussing their lifestyle<br>10.8% had unanswered questions	High
Trivedy 2016[12]	Journal article	Outreach NHS Health Check clinics at cricket grounds	513	Participants were asked to complete an anonymous questionnaire immediately after their NHS Health Check	Not given	83% rated their experience as excellent<br>100% would recommend to others	Medium

For peer review only

For peer review only - http://bmjopen.bmj.com/site/about/guidelines.xhtml

Table 2. Features of qualitative studies describing patient experiences of NHS Health Checks

Author, year	Type of report	Study period	Location of study	Setting of NHS Health Check	Data collection method	*n*	Method of recruitment to study	Participant characteristics	Overall quality
Alford 2010[25]	Evaluation report	Not given	Knowsley	Community	Interviews and focus groups	36	No details given	19 female, 17 male<br>13 high risk score, 23 low risk score	Medium
Baker 2014[10]	Journal article	2012	Gloucester	83 general practices	Content analysis of cross-sectional survey	1,011 (43%)	Survey sent to all patients who had completed an NHS Health Check within a 2 month period	55.2% female<br>19% 56-60 years<br>10.8% 40-45 years<br>96% white British	High
Chipchase 2011[26]	Report	2011	East and North Birmingham	2 general practices	Face-to-face semi-structured interviews	10	Attendees to NHS Health Checks in the first two weeks of February 2011 received a recruitment letter	8 female, 2 male	High
Corlett 2015[17]	Journal article	2013	London	4 pharmacies	Telephone interviews with sample of survey respondents	19	Invitation for a semi-structured telephone interview included with survey sent to all those who had attended an NHS Health Check within a 4 week period	Not given	Medium
Greenwich 2011[13]	Report	2011	Greenwich	Community	Open ended questionnaire, focus groups and in-depth phone interviews	612 survey responses<br>4 focus groups and 31 interviews	Recruited from community outreach services providing NHS Health Checks	Ethnic minority participants: 42% female	Medium
Ismail and Atkin 2015[18]	Journal article	Not given	Not specified	General practices	Semi-structured interviews	45 baseline<br>38 follow-up	Purposive sampling from a list provided by 5 participating general practices	21 female, 24 male.<br>Average age: 58.<br>Ethnicity: 37 White, 5 South Asian and 3 African Caribbean	High
Jenkinson 2015[19]	Journal article	2013	Torbay	4 general practices	Telephone or face-to-face interviews	17	Letters of invitation sent to a random sample identified by general practices from lists stratified by age and gender of those who had not responded to an invitation to an NHS Health Check within 4 weeks.	12 females, 5 males<br>6 employed, 1 unemployed, 10 retired	High
Krska 2015[9]	Journal	2011	Sefton, an	16 general	Postal survey	434	All patients with estimated 10 year	19% female	Medium

For peer review only - http://bmjopen.bmj.com/site/about/guidelines.xhtml

Author, year	Type of report	Study period	Location of study	Setting of NHS Health Check	Data collection method	*n*	Method of recruitment to study	Participant characteristics	Overall quality
	article		area of North West England	practices	with free text responses	(23.4%)	CVD risk > 20% from the 16 practices were sent a postal survey regardless of whether they had attended an NHS Health Check or not	68.2% over 65<br>99.5% white<br>7.7% highest quintile of deprivation<br>13.7% lowest quintile	
McNaughton 2015[29]	Journal article	2009-12	North East of England (non-specific location)	5 general practices	Semi-structured interviews	29	Invitations to patients from five general practices who had received an NHS Health Check and had an estimated 10 year CVD risk >20%	10 females, 19 males<br>24 over 65 years<br>13 in least deprived quintile	High
Oswald 2010[27]	Evaluation report	2009 - 2010	Teesside	General practices or pharmacies	Semi-structured interviews	8	Invited by general practices or pharmacies or from a list of patients who had attended an NHS Health Check and agreed to take part in the service evaluation	6 had attended general practices and 2 pharmacies	Medium
Perry 2014[21]	Journal article	2010	Knowsley	Community	Interviews and focus groups	36	Letter or telephone invitation to all 38 people who were at high risk of CVD and had attended an NHS Health Check in the past 12-18 months were invited. The remaining attendees at low risk of CVD were purposively sampled for gender, age , risk score.	3 focus groups: 1 for high risk scores (6 males), 2 for low risk scores (17 females and 7 males)<br>6 semi-structured interviews (2 females and 4 males with high risk score)	High
Riley 2015[23]	Journal article	2013	Bristol inner-city	Community	Semi-structured interviews	16	Participants were recruited via their attendance of community outreach events.	7 females, 9 males<br>All from black and minority ethnic populations	High
Riley 2015[22]	Journal article	2013-14	Bristol	General practices	Face-to-face and telephone semi-structured interviews	28	Purposive sampling from those identified through a search of patient records for patients who had undertaken an NHS Health Check within the previous 6 months	16 females, 12 males<br>23 White British<br>11 most deprived quintile<br>11 high (>20%) CVD risk	High
Shaw 2015[24]	Journal article	2010-11	Birmingham and Black Country	General practices and community	Semi-structured interviews	23	Patients who had attended an NHS Health Check were invited by practice managers or lead clinicians	High black and minority ethnic population and high levels of deprivation	High
Strutt 2011[28]	Masters thesis	2010	Darlington, Co. Durham, UK	2 general practices	Semi-structured face-to-face interviews	16	Invitation letters or telephone	7 females, 9 males<br>White, South-Asian, and Middle Eastern	High

For peer review only - http://bmjopen.bmj.com/site/about/guidelines.xhtml

Table 3. Studies contributing to each of the qualitative themes

	Unmet expectations	Limited understanding of the risk score	Preference for better information	Potential trigger for behaviour change	Confusion around follow-up
Alford 2010[25]			●	●	●
Baker 2014[10]	●	●	●	●	
Chipchase 2011[26]	●	●	●	●	
Corlett 2015[17]		●	●	●	
Greenwich 2011[13]			●	●	●
Ismail and Atkin 2015[18]	●	●		●	●
Jenkinson 2015[19]		●			
Krska 2015[9]	●	●			
McNaughton 2015[29]			●	●	
Oswald 2010[27]	●				●
Perry 2014[21]		●	●	●	
Riley 2015[23]		● (a)	●	●	
Riley 2015[22]	● (b)				
Shaw 2015[24]		●	●	●	●
Strutt 2011[28]	●	●	●	●	

Box 1. Reasons provided by participants for not making lifestyle changes

- Older participants feeling that making changes to their lifestyle was unnecessary [29]
- Healthy eating information was too generic [29]
- Guidance they had been given was likely to be subject to change [29]
- Co-morbidities which made physical activity difficult [29]
- Psychosocial circumstances, e.g. bereavement, stress or socio-economic barriers, such as shift work or unemployment [21,22]
- Having previously been offered a behaviour intervention strategy [18]
- Cost of eating fresh fruit and vegetables [18]
- Difficulty incorporating changes into their daily lives [21]
- Underlying medical conditions [21]

For peer review only

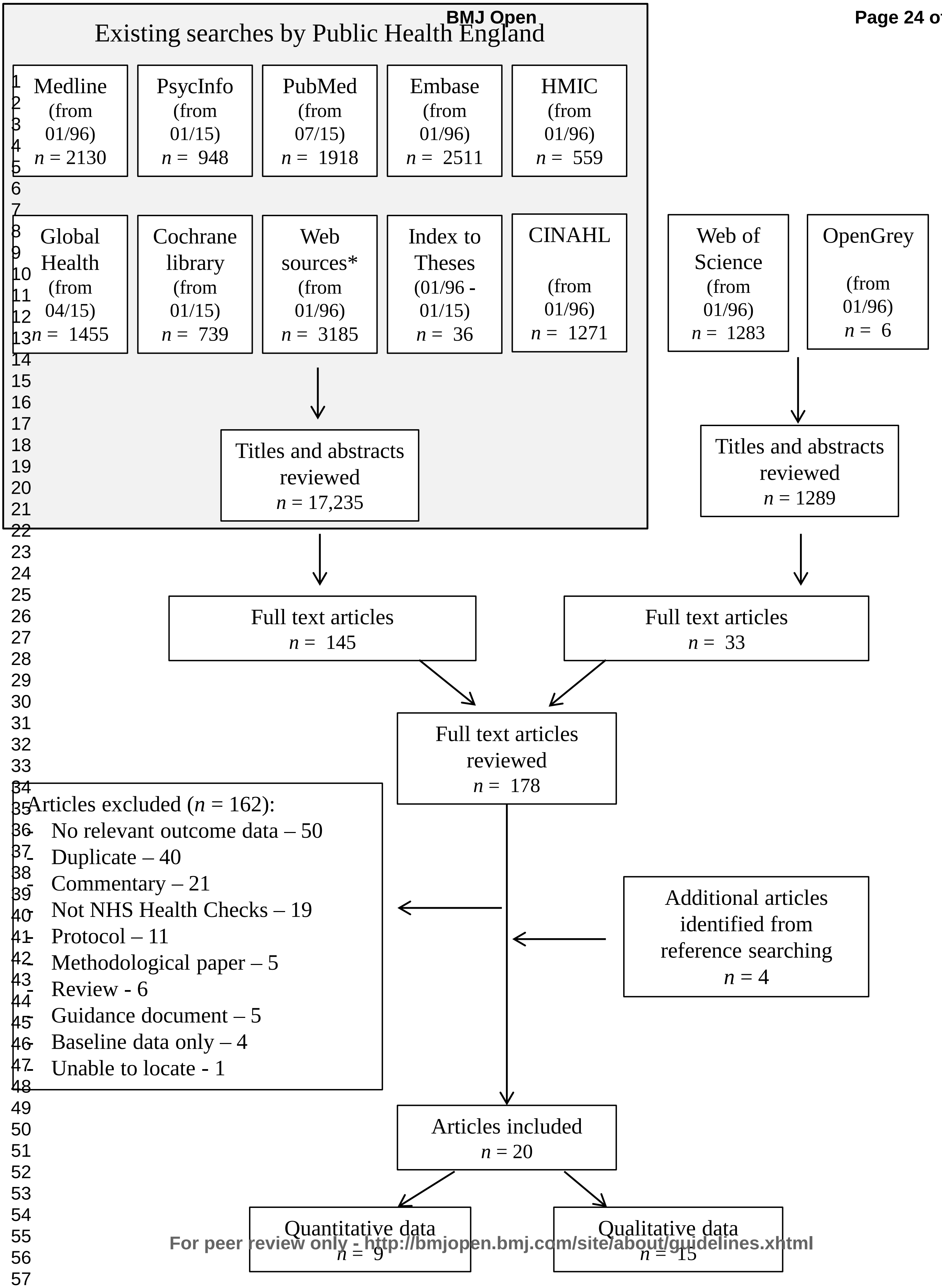
Existing searches by Public Health England
Medline (from 01/96) n = 2130
PsycInfo (from 01/15) n = 948
PubMed (from 07/15) n = 1918
Embase (from 01/96) n = 2511
HMIC (from 01/96) n = 559
Global Health (from 04/15) n = 1455
Cochrane library (from 01/15) n = 739
Web sources* (from 01/96) n = 3185
Index to Theses (01/96 - 01/15) n = 36
CINAHL (from 01/96) n = 1271
Web of Science (from 01/96) n = 1283
OpenGrey (from 01/96) n = 6
Titles and abstracts reviewed n = 17,235
Titles and abstracts reviewed n = 1289
Full text articles n = 145
Full text articles n = 33
Full text articles reviewed n = 178
Articles excluded (n = 162):
- No relevant outcome data – 50
- Duplicate – 40
- Commentary – 21
- Not NHS Health Checks – 19
- Protocol – 11
- Methodological paper – 5
- Review - 6
- Guidance document – 5
- Baseline data only – 4
- Unable to locate - 1
Additional articles identified from reference searching n = 4
Articles included n = 20
Quantitative data n = 9
Qualitative data n = 15

Appendix 1 – Search strategies

Database	Search strategy
Ovid Medline	1. health check*.tw.<br>2. (diabetes adj3 screen*).tw.<br>3. (cardiovascular adj3 screen*).tw.<br>4. (population adj2 screen*).tw.<br>5. (risk factor adj3 screen*).tw.<br>6. (opportunistic adj3 screen*).tw.<br>7. medical check*.tw.<br>8. general check*.tw.<br>9. periodic health exam*.tw.<br>10. annual exam*.tw.<br>11. annual review*.tw.<br>12. NHSHC.tw.<br>13. 1 or 2 or 3 or 4 or 5 or 6 or 7 or 8 or 9 or 10 or 11 or 12<br>14. cardiovascular adj3 prevention.tw.<br>15. (primary care or general practice or primary healthcare).tw<br>16. 14 and 15<br>17. Cardiovascular Diseases/ AND Primary Prevention/<br>18. 16 or 17<br>19. 13 or 18
PubMed	1. health check*<br>2. diabetes screen*<br>3. cardiovascular screen*<br>4. population screen*<br>5. risk factor screen*<br>6. opportunistic screen*<br>7. medical check*<br>8. general check*<br>9. periodic health exam*<br>10. annual exam*<br>11. annual review*<br>12. NHSHC<br>13. 1 or 2 or 3 or 4 or 5 or 6 or 7 or 8 or 9 or 10 or 11 or 12<br>14. Cardiovascular Diseases AND Primary Prevention[MeSH Terms]<br>15. "primary care"[Text Word] OR "general practice"[Text Word] OR "primary healthcare"[Text Word])<br>16. (cardiovascular[Text Word] AND prevention[Text Word])<br>17. #15 and #16<br>18. #14 or #17<br>19. #13 or #18
Ovid Embase	1. health check*.tw.<br>2. (diabetes adj3 screen*).tw.<br>3. (cardiovascular adj3 screen*).tw.<br>4. (population adj2 screen*).tw.

For peer review only

	5. (risk factor adj3 screen*).tw.<br>6. (opportunistic adj3 screen*).tw.<br>7. medical check*.tw.<br>8. general check*.tw.<br>9. periodic health exam*.tw.<br>10. annual exam*.tw.<br>11. annual review*.tw.<br>12. NHSHC.tw.<br>13. periodic medical examination/<br>14. 1 or 2 or 3 or 4 or 5 or 6 or 7 or 8 or 9 or 10 or 11 or 12 or 13<br>15. cardiovascular adj3 prevention.tw.<br>16. (primary care or general practice or primary healthcare).tw<br>17. 15 and 16<br>18. cardiovascular disease/ AND primary prevention/<br>19. 17 or 18<br>20. 14 or 19
Ovid HMIC	1 "health check*".af.<br>2 health checks/<br>3 (cardiovascular or vascular or heart or diabetes or stroke).af.<br>4 (screen* or risk).af.<br>5 3 AND 4<br>6 1 OR 2 or 5<br>7 cardiovascular adj3 prevention.tw.<br>8 (primary care or general practice or primary healthcare).tw<br>9 7 and 8<br>10 Cardiovascular diseases/ AND exp preventive medicine/<br>11 9 or 10<br>12 6 or 11
EBSCO CINAHL	S10 S1 OR S2 OR S9<br>S9 S5 OR S8<br>S8 S6 AND S7<br>S7 (MH "Preventive Health Care+")<br>S6 (MH "Cardiovascular Diseases+")<br>S5 S3 AND S4<br>S4 "primary care" or "general practice" or "primary healthcare"<br>S3 TX cardiovascular N3 prevention<br>S2 (diabetes N3 screen*) OR (cardiovascular N3 screen*) OR (population N2 screen*) OR (risk factor N3 screen*) OR (opportunistic N3 screen*) OR “medical check*” OR “general check*” OR “periodic health exam*” OR “annual exam*” OR "annual review*" OR NHSHC<br>S1 health check*
EBSCO Global Health	S10 S6 OR S19 OR S3 Limiters - Publication Year: 2016<br>S9 S7 AND S8<br>S8 DE "preventive medicine"<br>S7 DE "cardiovascular diseases"<br>S6 S4 AND S5<br>S5 "primary care" or "general practice" or "primary healthcare"

For peer review only

	S4 TX cardiovascular N3 prevention<br>S3 S1 OR S2<br>S2 (diabetes N3 screen*) OR (cardiovascular N3 screen*) OR (population N2 screen*) OR (risk factor N3 screen*) OR (opportunistic N3 screen*) OR “medical check*” OR “general check*” OR “periodic health exam*” OR “annual exam*” OR "annual review*" OR NHSHC<br>S1 health check*
HDAS PsycInfo	1 "health check*".af.<br>2 PHYSICAL EXAMINATION/<br>3 HEALTH SCREENING/<br>4 "diabetes screen*".af<br>5 "cardiovascular screen*".af<br>6 "population screen*".af<br>7 ("opportunistic* screen*" OR "risk factor screen*").af<br>8 ("medical check*" OR "general check*" OR "periodic health exam*" OR "annual exam*" OR "annual review*" OR NHSHC).af<br>9 1 OR 2 OR 3 OR 4 OR 5 OR 6 OR 7 OR 8<br>10 cardiovascular.ti,ab<br>11 prevention.ti,ab<br>12 10 AND 11<br>13 CARDIOVASCULAR DISORDERS/<br>14 PREVENTIVE MEDICINE/<br>15 13 AND 14<br>16 12 OR 15<br>17 9 OR 16
Web of Science, Science Citation Index	“health check*” OR “diabetes screen*” OR “cardiovascular screen*” OR “population screen*” OR “risk factor screen*” OR “Opportunistic screen*” OR “medical check*” OR “general check*” OR “periodic health exam*” OR “annual exam*” OR “annual review*” OR NHSHC<br>OR<br>(Cardiovascular NEAR/3 prevention) AND (“primary care” OR “general practice” OR “primary healthcare”)<br>Limit to: England, Scotland, Wales, North Ireland
Cochrane Library (Wiley)	#1 "health check*"<br>#2 (diabetes next/3 screen*) or (cardiovascular next/3 screen*) or (population next/2 screen*) or (opportunistic next/2 screen*) or ("risk factor" next/3 screen*) or "medical check*" or "general check*" or "periodic health exam*" or "annual exam*" or "annual review*" or NHSHC<br>#3 cardiovascular adj3 prevention.tw.<br>#4 (primary care or general practice or primary healthcare).tw<br>#5 #3 and #4<br>#6 MeSH descriptor: [Cardiovascular Diseases] this term only<br>#7 MeSH descriptor: [Primary Prevention] explode all trees<br>#8 #6 and #7<br>#9 #5 or #8<br>#10 #1 or #2 or #9

For peer review only

NHS Evidence	“health check*” OR cardiovascular prevention primary care
TRIP database	“health check*” OR cardiovascular prevention primary care
Google Scholar	"nhs health check"<br>cardiovascular “health check”<br>cardiovascular prevention “primary care”
Google	"nhs health check"<br>cardiovascular prevention “primary care”<br>cardiovascular “health check”
Clinical trials.gov and ISRCDN registry	“health check”

For peer review only

Appendix 2 – Quality assessment of quantitative studies

Author, date	Study addressed a clearly focused issue	Use of an appropriate method	Recruitment	Exposure measurement	Outcome measurement	Confounding factors	Applicability to England	Overall
Baker 2014	High	Medium	High	Medium	Medium	Low	Medium	High
Corlett 2015	High	High	High	High	High	Low	Low	High
Cowper 2013	High	Low	Low	Low	Low	Low	Medium	Low
Krska 2015	High	High	High	Medium	High	Low	Medium	High
LGA – East Riding 2015	High	Low	Low	Low	Low	Low	Low	Low
NHS Greenwich	Medium	Medium	Medium	Medium	Medium	Low	Low	Medium
'A picture of Health'	Medium	Low	Low	Low	Low	Low	Low	Low
Taylor 2012	High	High	Medium	High	High	Low	Medium	High
Trivedy 2016	Medium	Medium	Medium	High	Medium	Low	Low	Medium

• Low ● Medium ⬤ High

For peer review only

Appendix 3 – Quality assessment of qualitative studies

Author and date	Study addressed a clearly focused issue	Appropriateness of qualitative method	Design	Recruitment	Consideration of relationship between research and participants	Ethical issues	Rigor of data analysis	Clarity of statement of findings	Overall
Alford 2010	High	High	High	Low	Low	Low	Low	Medium	Medium
Baker 2014	High	High	High	High	n/a	Low	High	High	High
Chipchase 2011	High	High	High	Medium	Medium	High	High	Medium	High
Corlett 2015	High	High	High	Medium	Low	Medium	Medium	Medium	Medium
Greenwich 2011	High	High	High	Medium	Low	Low	Low	Medium	Medium
Ismail and Atkin 2015	High	High	High	High	Medium	High	High	High	High
Jenkinson 2015	High	High	High	High	Low	High	High	High	High
Krska 2015	High	High	Medium	High	n/a	Medium	Medium	Medium	Medium
McNaughton 2015	High	High	High	High	Low	High	High	High	High
Oswald 2010	High	High	High	Medium	Low	Medium	Medium	Medium	Medium
Perry 2014	High	High	High	High	Low	Low	High	High	High
Riley 2015 (JPH)	High	High	High	Medium	Medium	High	High	High	High
Riley 2015 (BMC HSR)	High	High	High	Medium	Medium	High	High	High	High
Shaw 2015	High	High	High	Medium	Low	High	High	High	High
Strutt 2011	High	High	High	High	High	High	High	High	High

• Low ● Medium ⬤ High

For peer review only

PRISMA 2009 Checklist

Section/topic	#	Checklist item	Reported on page #
**TITLE**			
Title	1	Identify the report as a systematic review, meta-analysis, or both.	1
**ABSTRACT**			
Structured summary	2	Provide a structured summary including, as applicable: background; objectives; data sources; study eligibility criteria, participants, and interventions; study appraisal and synthesis methods; results; limitations; conclusions and implications of key findings; systematic review registration number.	2
**INTRODUCTION**			
Rationale	3	Describe the rationale for the review in the context of what is already known.	4
Objectives	4	Provide an explicit statement of questions being addressed with reference to participants, interventions, comparisons, outcomes, and study design (PICOS).	4
**METHODS**			
Protocol and registration	5	Indicate if a review protocol exists, if and where it can be accessed (e.g., Web address), and, if available, provide registration information including registration number.	5
Eligibility criteria	6	Specify study characteristics (e.g., PICOS, length of follow-up) and report characteristics (e.g., years considered, language, publication status) used as criteria for eligibility, giving rationale.	5
Information sources	7	Describe all information sources (e.g., databases with dates of coverage, contact with study authors to identify additional studies) in the search and date last searched.	5
Search	8	Present full electronic search strategy for at least one database, including any limits used, such that it could be repeated.	Appendix 1
Study selection	9	State the process for selecting studies (i.e., screening, eligibility, included in systematic review, and, if applicable, included in the meta-analysis).	5
Data collection process	10	Describe method of data extraction from reports (e.g., piloted forms, independently, in duplicate) and any processes for obtaining and confirming data from investigators.	5/6
Data items	11	List and define all variables for which data were sought (e.g., PICOS, funding sources) and any assumptions and simplifications made.	Table 1 and 2
Risk of bias in individual studies	12	Describe methods used for assessing risk of bias of individual studies (including specification of whether this was done at the study or outcome level), and how this information is to be used in any data synthesis.	6
Summary measures	13	State the principal summary measures (e.g., risk ratio, difference in means).	6
Synthesis of results	14	Describe the methods of handling data and combining results of studies, if done, including measures of consistency (e.g., I^2) for each meta-analysis.	6

For peer review only - http://bmjopen.bmj.com/site/about/guidelines.xhtml

PRISMA 2009 Checklist

Section/topic	#	Checklist item	Reported on page #
Risk of bias across studies	15	Specify any assessment of risk of bias that may affect the cumulative evidence (e.g., publication bias, selective reporting within studies).	5
Additional analyses	16	Describe methods of additional analyses (e.g., sensitivity or subgroup analyses, meta-regression), if done, indicating which were pre-specified.	N/A
**RESULTS**			
Study selection	17	Give numbers of studies screened, assessed for eligibility, and included in the review, with reasons for exclusions at each stage, ideally with a flow diagram.	6 and Fig 1
Study characteristics	18	For each study, present characteristics for which data were extracted (e.g., study size, PICOS, follow-up period) and provide the citations.	Table 1 and 2
Risk of bias within studies	19	Present data on risk of bias of each study and, if available, any outcome level assessment (see item 12).	Table 1 and 2 and Appendix 2
Results of individual studies	20	For all outcomes considered (benefits or harms), present, for each study: (a) simple summary data for each intervention group (b) effect estimates and confidence intervals, ideally with a forest plot.	N/A
Synthesis of results	21	Present results of each meta-analysis done, including confidence intervals and measures of consistency.	N/A
Risk of bias across studies	22	Present results of any assessment of risk of bias across studies (see Item 15).	N/A
Additional analysis	23	Give results of additional analyses, if done (e.g., sensitivity or subgroup analyses, meta-regression [see Item 16]).	N/A
**DISCUSSION**			
Summary of evidence	24	Summarize the main findings including the strength of evidence for each main outcome; consider their relevance to key groups (e.g., healthcare providers, users, and policy makers).	11
Limitations	25	Discuss limitations at study and outcome level (e.g., risk of bias), and at review-level (e.g., incomplete retrieval of identified research, reporting bias).	12
Conclusions	26	Provide a general interpretation of the results in the context of other evidence, and implications for future research.	13/14
**FUNDING**			
Funding	27	Describe sources of funding for the systematic review and other support (e.g., supply of data); role of funders for the systematic review.	14/15

For peer review only - http://bmjopen.bmj.com/site/about/guidelines.xhtml

PRISMA 2009 Checklist

From: Moher D, Liberati A, Tetzlaff J, Altman DG, The PRISMA Group (2009). Preferred Reporting Items for Systematic Reviews and Meta-Analyses: The PRISMA Statement. PLoS Med 6(6): e1000097. doi:10.1371/journal.pmed1000097

For more information, visit: www.prisma-statement.org.

For peer review only

For peer review only - http://bmjopen.bmj.com/site/about/guidelines.xhtml

BMJ Open

Patient experience of NHS Health Checks: a systematic review and qualitative synthesis

Journal:	*BMJ Open*
Manuscript ID	bmjopen-2017-017169.R1
Article Type:	Research
Date Submitted by the Author:	05-May-2017
Complete List of Authors:	Usher-Smith, Juliet; University of Cambridge, The Primary Care Unit<br>Harte, Emma; RAND Europe<br>MacLure, Calum; RAND Europe<br>Martin, Adam; Academic Unit of Health Economics, Leeds Institute of Health Sciences<br>Saunders, Catherine; University of Cambridge,<br>Meads, Catherine; Anglia Ruskin University, Faculty of Health, Social Care and Education<br>Walter, Fiona; University of Cambridge, Dept of Public Health and Primary Care<br>Griffin, Simon; University of Cambridge, The Primary Care Unit; University of Cambridge, MRC Epidemiology Unit<br>Mant, Jonathan; University of Cambridge, The Primary Care Unit
**Primary Subject Heading**:	Public health
Secondary Subject Heading:	Cardiovascular medicine, Communication, Qualitative research
Keywords:	NHS Health Check, patient experience, systematic review, qualitative synthesis

SCHOLARONE™
Manuscripts

Patient experience of NHS Health Checks: a systematic review and qualitative synthesis

Usher-Smith JA[1], Harte E[2], MacLure C[2], Martin A[3], Saunders CL[1], Meads C[4], Walter FM[1], Griffin SJ[1,5], Mant J[1].

[1]The Primary Care Unit, Institute of Public Health, University of Cambridge, Box 113 Cambridge Biomedical Campus, Cambridge, CB2 0SR, UK

[2]RAND Europe, Westbrook Centre, Milton Road, Cambridge, CB4 1YG, UK

[3]Academic Unit of Health Economics, Leeds Institute of Health Sciences, University of Leeds, Leeds, LS2 9LJ, UK

[4]Faculty of Health, Social Care and Education, Anglia Ruskin University

[5]MRC Epidemiology Unit, University of Cambridge, Institute of Metabolic Science, Cambridge, CB2 0QQ, UK

Correspondence to: Juliet Usher-Smith jau20@medschl.cam.ac.uk

Key words: NHS Health Check, patient experience, systematic review, qualitative synthesis

For peer review only

For peer review only - http://bmjopen.bmj.com/site/about/guidelines.xhtml

ABSTRACT (300 words)

Objective: To review the experiences of patients attending NHS Health Checks in England.

Design: A systematic review of quantitative and qualitative studies with a thematic synthesis of qualitative studies.

Data sources: An electronic literature search of Medline, Embase, Health Management Information Consortium (HMIC), Cumulative Index of Nursing and Allied Health Literature (CINAHL), Global Health, PsycInfo, Web of Science, OpenGrey, the Cochrane Library, NHS Evidence, Google Scholar, Google, Clinical Trials.gov and the ISRCTN registry to 09/11/16 with no language restriction and manual screening of reference lists of all included papers.

Inclusion criteria: Primary research reporting experiences of patients who have attended NHS Health Checks.

Results: 20 studies met the inclusion criteria, 9 reporting quantitative data and 15 qualitative data. There were consistently high levels of reported satisfaction in surveys, with over 80% feeling that they had benefited from an NHS Health Check. Data from qualitative studies showed that the NHS Health Check had been perceived to act as a wake-up call for many who reported having gone on to make substantial lifestyle changes which they attributed to the NHS Health Check. However, some had been left with a feeling of unmet expectations, were confused about or unable to remember their risk scores, found the lifestyle advice too simplistic and non-personalised, or were confused about follow-up.

Conclusions: Whilst participants were generally very supportive of the NHS Health Check programme and examples of behaviour change were reported, there are a number of areas where improvements could be made. These include greater clarity around the aims of the programme within the promotional material, more pro-active support for lifestyle change, and greater appreciation of the challenges of communicating risk and the limitations of relying on the risk score alone as a trigger for facilitating behaviour change.

For peer review only - http://bmjopen.bmj.com/site/about/guidelines.xhtml

Strengths and limitations of this study

- This is the first study to systematically review quantitative and qualitative studies that consider the experiences of patients who have attended NHS Health Checks.
- The use of broad inclusion criteria and the systematic search of multiple databases and the grey literature allowed us to include studies that had not been published in peer reviewed scientific journals.
- The included studies were of varying quality.
- The quantitative studies reporting responses to surveys had response rates between 23% and 43%, making them at risk of responder bias.
- The qualitative studies included small, selected groups of participants whose expressed views were likely to be affected by both recall bias and social desirability bias.

peer review only

For peer review only - http://bmjopen.bmj.com/site/about/guidelines.xhtml

INTRODUCTION

The NHS Health Check programme is one of the largest current prevention initiatives in England. Introduced in 2009 to improve cardiovascular disease (CVD) risk factors through behavioural change and treatment informed by risk stratification, it became a mandated public health service in 2013. Local authorities are now responsible for offering an NHS Health Check to individuals aged 40-74 without existing cardiovascular disease, diabetes or hypertension every five years. The NHS Health Check itself consists of three components: risk assessment, communication of risk and risk management[1]. For CVD the QRISK®2 risk tool[2] is first used to estimate the individual's risk of developing CVD based on risk factors including age, sex, ethnicity, smoking status, height and weight, family history of coronary heart disease, blood pressure, and cholesterol. That estimated risk, expressed as the percentage risk of developing disease over the next 10 years, is then used to raise awareness of relevant risk factors and inform discussion about the lifestyle and medical approaches best suited to managing the individual's risk of disease. Risk assessment for diabetes was introduced in 2016 and patients at high risk of developing type 2 diabetes who should receive a screening blood test are identified either by using either validated risk assessment tools or a diabetes filter[1]. Based on modelling studies of cross-sectional data it was estimated that the programme could prevent 1,600 heart attacks and strokes, at least 650 premature deaths, and over 4,000 new cases of diabetes each year with an estimated cost per quality adjusted life year (QALY) of approximately £3,000[3]. However, whether NHS Health Checks represent an efficient use of scarce health promotion resources has been questioned[4,5].

Alongside clinical effectiveness and safety, patient experience is increasingly recognised worldwide as one of the three elements of high-quality healthcare[6–8]. As well as enabling a better understanding of current problems with healthcare delivery, informing continuous improvement and redesign of services and helping professionals reflect on practice, a recent systematic review has shown that patient experience is positively associated with self-rated and objectively measured health outcomes; adherence to recommended medication and treatments; preventative care; healthcare resource use; technical quality-of-care delivery; and adverse events[9,10]. There is also an association at the organizational level; general practices that provide higher quality clinical care (measured through higher QOF performance) are also those in which reported patient experience is better. Understanding patients' experiences of NHS Health Checks is, therefore, central to understanding the implementation of the programme, its potential impact over the first eight years, and ways in which it might be

For peer review only - http://bmjopen.bmj.com/site/about/guidelines.xhtml

improved to increase adherence to lifestyle advice and preventive treatments and, ultimately, improve health outcomes.

Since the introduction of the NHS Health Check programme, a growing number of both quantitative and qualitative studies reporting patients' experiences of NHS Health Checks have been published. This article provides the first systematic synthesis of these studies.

METHODS

We performed a systematic literature review following a study protocol (available on request) that followed the PRISMA guidelines.

Search strategy

We used the results of an existing literature review conducted by Public Health England covering the period from 1st January 1996 to 9th November 2016 supplemented by a search of the Web of Science, Science Citation Index and OpenGrey covering the same period. We also hand searched the reference lists of all included publications, searched online for additional articles published by authors of the included studies, and contacted the NHS Health Checks Expert Scientific and Clinical Advisory Panel to identify studies in progress or near completion. The PHE literature review included the following sources: Medline, Embase, Health Management Information Consortium (HMIC), Cumulative Index of Nursing and Allied Health Literature (CINAHL), Global Health, PsycInfo, the Cochrane Library, NHS Evidence, Google Scholar, Google, Clinical Trials.gov and the ISRCTN registry. Full details of all the search strategies are shown in Appendix 1. No language restrictions were applied.

Study selection

To be included studies had to be primary research reporting the opinions or experiences of people who had attended NHS Health Checks. Commentaries, editorials and opinion pieces were excluded.

The selection of studies was performed in a two-stage process. First, the titles and abstracts were screened to identify studies relevant to the NHS Health Check. This stage had already been completed by a senior information scientist at PHE for those identified in the literature review conducted by PHE. One reviewer (EH) followed this process for the additional citations identified from the Web of Science database.

For peer review only - http://bmjopen.bmj.com/site/about/guidelines.xhtml

In the second stage two researchers (JUS and AM) reviewed the full texts of all studies identified as relevant to the NHS Health Checks to select those reporting the opinions or experiences of people who had attended NHS Health Checks. Where it was unclear whether or not these inclusion criteria were met for any given study, we discussed those studies at consensus meetings with the wider research team.

Data extraction, quality assessment and synthesis

Data on the study design, time period, recruitment methods, participants, analysis, and quantitative results were extracted independently for each study by two reviewers (JUS + EH/CMa) onto data extraction forms developed to minimise bias. The quality of all each included studies was assessed at the same time using the Critical Appraisal Skills Programmes (CASP)[11] checklist for qualitative research or a checklist combining the CASP checklists for cohort studies and randomised-controlled trials for the quantitative studies. We chose these checklists as they are included within the Cochrane Supplemental Guidance for Inclusion of Qualitative Research in Cochrane Systematic Reviews of Interventions[12] and identified as one of the 14 'best' tools for evaluating non-randomised quantitative studies in a review[13] respectively, and we have successfully used them in previous reviews[14,15]. For studies that included both quantitative and qualitative methods, quality assessment was completed separately for both aspects of the study. No studies were excluded on the basis of quality alone.

As a result of the variation in methods use and experiences reported, we were unable to perform meta-analysis for the quantitative data and so synthesised that data descriptively. We synthesised the qualitative data using thematic synthesis[16]. Following reading and re-reading of the included studies, this synthesis included three stages: 1) coding of the findings of the primary studies; 2) organisation of these codes into related areas to develop descriptive themes; and 3) the development of analytical themes. As described by Thomas and Harden[16], we considered all the text under the headings 'Results' or 'Findings' within the included studies as findings of the primary studies. The initial line-by-line coding of those findings was performed by at least two researchers (JUS + EH/CMa), each from a different disciplinary background (academic general practice, public services, and health systems and innovation). All have experience conducting and analysing qualitative research but none had been involved in any of the included studies. The codes resulting from that process were then

For peer review only

For peer review only - http://bmjopen.bmj.com/site/about/guidelines.xhtml

discussed with members of the wider research team and the subsequent stages were an iterative process with both the descriptive and analytical themes developed through a series of meetings involving researchers from a range of clinical and non-clinical backgrounds (academic general practice, public health, health economics, clinical statistics, evidence synthesis and qualitative research). To allow an appreciation of the primary data, we have included illustrative quotations from the original studies alongside the analytical themes in this report.

A summary of the findings reported in this manuscript have been published online by Public Health England (available at http://www.healthcheck.nhs.uk/commissioners_and_providers/evidence/) and RAND (http://www.rand.org/content/dam/rand/pubs/external_publications/EP60000/EP67129/RAND_EP67129.pdf). Permission from both has been obtained to publish the results in this journal.

RESULTS

From an initial 18,524 titles and abstracts, 178 articles were identified as potentially relevant to the NHS Health Checks and were reviewed at full text level (Figure 1). Of those, we excluded 162. The most common reasons for excluding papers were that they did not include any relevant data, were duplicates or commentaries, or did not describe NHS Health Checks. Four additional articles were identified through citation searching. This review is, therefore, based on 20 articles.

Quantitative results from patient satisfaction questionnaires

Of those 20 articles, nine include quantitative results from surveys of participants who had attended NHS Health Checks[17–25]. The details of these nine articles are shown in Table 1 and full details of the quality assessment in Appendix 2. Four are high quality journal articles published in peer reviewed journals in which questionnaires were sent to all those who had attended an NHS Health Check in either general practices[17,18] or pharmacies[24,25]. Response rates were between 23.4% and 43%. A fifth study of the views of those attending outreach clinic at cricket groups was also published in a peer reviewed journal but does not report the methods in detail or the response rate[20]. Another is a report of a service evaluation in which

For peer review only - http://bmjopen.bmj.com/site/about/guidelines.xhtml

the views of ethnic minority participants were particularly sought[21] and the final three are low quality case study reports which have not reported methods or response rate[19,22,23].

The findings from those nine studies are summarised in Table 2. Eight included questions about the overall experience and satisfaction with attending an NHS Health Check. Over 80% of respondents rated the experience highly or reported high levels of satisfaction. Between 86% and 99% also felt they had benefited from the NHS Health Check or would be likely or very likely to return if invited back and over 78% would recommend attendance to others. When reported (*n*=4), 88% to 99% of respondents felt they were given enough time. However, between 7% and 15% still had unanswered questions after the NHS Health Check.

.

Qualitative data on patient experience

Patient experience was also reported in 15 qualitative studies. Three performed content analysis on free-text responses provided in surveys[17,18,21] whilst the others conducted focus groups or interviews with between 8 and 45 participants. Ten are journal articles published in peer reviewed journals[17,18,25–32] four are research reports of service evaluations[21,33–35], and one is a Masters thesis[36]. All recruited people who had attended NHS Health Checks either through invitations sent out from general practices or from community settings. Most included approximately equal numbers of men and women. Three studies had particularly sought to describe the experiences of those from ethnic minority groups[21,31,32]. In the quality assessment, ten were high quality and five medium quality, with all addressing a clearly focused issue and using an appropriate qualitative method and design. The reflexivity showed the greatest variation across the studies with only five scoring medium or high for consideration of the relationship between the research team and participants. Most analysed data using thematic analysis. Further details of the design and methods used in those studies are given in Table 3 and the full quality assessment in Appendix 3.

Thematic synthesis of these 15 studies identified five main analytical themes: 1) The NHS Health Check as a potential trigger for behaviour change; 2) Unmet expectations; 3) Limited understanding of the risk score; 4) Preference for better information; and 5) Confusion around follow-up. The primary articles contributing to each of those themes are shown in Table 4 and details of each of the themes given below.

For peer review only - http://bmjopen.bmj.com/site/about/guidelines.xhtml

1. The NHS Health Check as a potential trigger for behaviour change

Participants variously described the NHS Health Check as a "*wake-up call"*[21,29], a "*reality check*"[29], a "*kind of a turning point"*[36], or an "*eye-opener"*[32], which helped bring patients' health into focus by highlighting potential underlying health issues of which they were not necessarily cognizant[18] and making them aware that there were lifestyle-related diseases to which they may be susceptible and which they may be able to prevent[32,34].

> "*It's really good. It makes you aware of what problems are around. What you can get and that. It is really good. It teaches you..it's an eye-opener for people who would want to do things properly*" [32]

For some, this reputedly led on to behaviour change, with many of the studies citing examples of participants who had reported making changes that they attributed to having attended the NHS Health Check[26,29–33,37]. These included changes to diet, cutting down on smoking, decreasing alcohol intake and increasing physical activity.

> *"I've changed my diet um and, and lost a stone in weight I think as a result actually. So I'm quite happy with that, that makes me feel even healthier"* [30]

> "*Having the results of the check, I've actually started to go to [swimming baths] a couple of times, so I've made some progress….and I've actually felt better in meself.*" [29]

In general, dietary changes were perceived to be the easiest changes to make, particularly small changes that did not cause too much disruption to their daily routines[37] and there was recognition that changing behaviour was hard, with a number of barriers identified (Box 1).

2. Unmet expectations

Despite this potential influence on behaviour, a strong theme throughout many of the studies was that of unmet expectations that some participants were left with at the end of the NHS Health Check.

For many, this arose from confusion about the purpose of the NHS Health Check. The comparison made between the NHS Health Check and an 'MOT' in the promotional material

For peer review only - http://bmjopen.bmj.com/site/about/guidelines.xhtml

and the use of the term 'Health Check' left many expecting the NHS Health Check to include a more general wide-ranging assessment of health and not just risk of cardiovascular disease[18,26,34].

> "*I just assumed that they would test you for everything when you were there. My perception of reading through things was that it was going to be a good overhaul, you know overall body check for everything.*" [34]

> "*As a general health check it was not a series of tests as I expected. Only centred around the result of a blood test. Not comprehensive as I would have expected*" [17]

Additional specific areas that participants had been expecting or thought should be covered included: a Well woman check[34]; diabetes checks for all[31,34,35]; cancer screening[26,34,36]; an assessment of mental well-being[26]; an ECG[26]; testing for anaemia[26]; discussion around health conditions that impacted on their daily lives, such as joint and back pain[36]; and chronic long-term conditions[35].

3. Limited understanding of the risk score

Whilst some participants reported improved understanding of CVD risk following an NHS Health Check[17,25,32], a common theme throughout the studies was participants' limited understanding of the risk score.

Across many of the studies there was evidence that a large number of participants were either not able to recall being provided with a risk estimate at all[17,26,27], found the risk score confusing[18,30,34], or had interpreted it incorrectly[26,29,30,36].

> "*my cholesterol is high…and, I had a score saying sixteen per cent diabetes in ten years. What does that mean? I've got no idea what that means. It sounds bad because it's higher than it's meant to be but is it?*"[30]

> "'*My QRisk score is 11 per cent. But after getting someone to Google it for me, we still have no idea what it means. It should be explained better in a letter from the Doctor*" [18]

For peer review only - http://bmjopen.bmj.com/site/about/guidelines.xhtml

The score itself also appeared to have little meaning or significance for most participants. Low scores (<20% 10-year estimated risk) were sometimes perceived as meaning there was nothing to worry about[29], but participants with low-risk scores were as likely to report being worried or anxious after receiving the scores as those with high-risk scores[30]. When describing their motivation to change behaviour, in general participants also described how it was not necessarily related to their risk score, and how even a high risk score was not necessarily enough to motivate them to try and change[29,36].

> "*Sometimes you need a reason and I think it was like me, I needed a reason [to change] and isn't it sad that showing me the percentage wasn't reason enough for me to give up [smoking].*" [29]

4. Preferences for better information

Most participants reported receiving lifestyle advice within the NHS Health Check. Many, however, felt it was too simple, brief, superficial or generic and felt that they would have benefitted from more detail and more personalised information[21,25,29,30,32,36,37].

> *"And it was that kind of information which was the kind of the bit beyond, you know, eat less, exercise more, don't smoke, don't drink .. .that would have been useful ..the kind of advice that was on offer was actually very, um, simple"* [30]

For some this lack of personalised information led to confusion and uncertainty[36], with some feeling that they had received mixed messages about their health[26] and been left unsure about what actions they should take[33]. This was not a universal view, however, with some seeing the value in being provided with 'common knowledge' again as it afforded a fresh way of looking at their lifestyle and, in one study the simplicity of the information appeared to encourage participants to make changes to their behaviour[29].

> *"So I thought it was very helpful it was very informative and it was thought-provoking, it just gave us some fresh view on things, because you can get very easily into doing what you think is okay"* [36]

In most cases the lifestyle advice had been provided face-to-face but participants also valued, or felt it would have been helpful to have received, written information both for their own

For peer review only - http://bmjopen.bmj.com/site/about/guidelines.xhtml

reference and also as a means to encourage behaviour change among friends and family[18,29,34].

> *"Well I suppose it's good to have a question and answer thing cos you can have somebody explain it to you. But I suppose you could, something written'd be quite useful."* [26]

5. Confusion around follow-up

The final theme related to confusion over follow-up[21,32,33]. This was particularly seen amongst participants who had attended NHS Health Checks in community settings. Individuals felt unsure about what steps should be taken next, specifically in relation to whether they needed to contact their GP or if their GP would contact them if any causes for concern had been identified[33]. Participants also reported a lack of sufficient information on follow-up and sign-posting to other NHS services[21].

> *"She never said go and see your doctor. She ran out of some leaflets and she circled them and said go on the Internet"* [21]

Some participants also reported that they would have liked their healthcare professionals to be more pro-active in supporting them to make lifestyle changes and felt there should have been on-going follow-up and monitoring[26,32,35].

DISCUSSION

Principal findings

This study is the first systematic review of patient experience of NHS Health Checks. It shows that, amongst those who respond to patient satisfaction surveys, there are consistently very high levels of satisfaction with NHS Health Checks reported, with over 80% feeling that they had benefited from an NHS Health Check. However, despite these overall high levels of satisfaction, there was evidence from interviews that some participants were left with a feeling of unmet expectations. For some this appeared to arise from confusion about the purpose of the NHS Health Check whilst others had been expecting a more general assessment of health. The cardiovascular risk score also appeared to generate confusion: it was poorly understood, interpreted differently among individuals with the same level of risk, and seemed to have little meaning or significance for people in terms of how to use it to think

For peer review only - http://bmjopen.bmj.com/site/about/guidelines.xhtml

For peer review only

about their health and future planning. Most participants reported receiving lifestyle information within the NHS Health Check but for many it was regarded as too simple and not sufficiently personalised. Nevertheless, there was evidence that the NHS Health Check was perceived to act as a wake-up call for many participants who had gone on to make lifestyle changes which they attributed to the NHS Health Check.

Strengths and Limitations

The main strengths of this review are the systematic search of multiple electronic databases, the manual searching of the reference lists of all included studies, and the thematic approach to synthesis of the qualitative data. By including searches of the grey literature and the internet alongside electronic repositories we were able to include studies that have not been published in peer reviewed scientific journals, reducing the risk of selective reporting bias. Whilst we cannot exclude the possibility that additional local evaluations may have been performed or that we overlooked studies at the screening stage, we think it unlikely that they would substantially alter the main findings. Choosing to conduct a thematic synthesis for the qualitative research also ensured that we used a systematic approach to identify common themes across the studies and interpret those findings. Although some argue against the synthesis of qualitative research on the grounds that the findings of individual studies are de-contextualised and the concepts identified in one setting are not applicable to another[38], the systematic approach to coding and subsequent development of overarching themes guided by our research question enabled us not only to provide a synthesis of the evidence to inform practice but also to develop additional interpretations and conceptual insights beyond the findings of the primary studies.

The main limitations relate to the included studies. The quantitative surveys were of varying quality with response rates only reported in four of the nine studies and comparison between responders and non-responders only in one. Where reported, the response rates varied between 23% and 43%. Although survey response rates alone are a poor indicator of bias[39,40], the included studies are, therefore, all at risk of responder bias, and may represent the views of those more engaged with preventive healthcare or with particularly strong opinions. Many also measured patient satisfaction rather than patient experience. Unlike patient experience data which aim to avoid value judgments, patient satisfaction is a broad and often ill-defined concept that is multi-dimensional and influenced by a range of factors, including cultural norms, health status and prior experience of health care[41]. Reports of patient satisfaction can,

For peer review only - http://bmjopen.bmj.com/site/about/guidelines.xhtml

therefore, vary widely between different patients in identical situations and in one study in which patients were asked a single question about how satisfied overall they were with their primary care practice, only 4.6% of the variance in their satisfaction ratings was a result of differences between practices[42]. Unlike patient experience data, improvements in patient satisfaction data are also not associated with improvements in care quality[43].

The qualitative studies also included small, selected groups of participants whose expressed views are likely to be affected by both recall bias (systematic errors due to inaccuracy of recollections about NHS Health Checks) and social desirability bias (the tendency of interviewees to give responses that they think might be viewed favourably by the interviewer)[44]. By virtue of the fact that they have chosen to take part in medical research the participants may also be more interested in their health than the general population so their views may not reflect the full range of views and experiences of those attending NHS Health Checks.

Comparison with existing literature

The high levels of satisfaction reported are consistent with those reported for other NHS services, for example the General Practice Patient Survey in which the median overall satisfaction score was 86.2[10]. The discrepancy between the very high levels of reported satisfaction in surveys and the more negative comments made in face-to-face interviews is also consistent with previous research in other areas of health care[45–48]. For example, studies have found that positive survey responses can mask important negative dimensions which patients subsequently express qualitatively[45–47] and that patients may respond differently to questions about services depending on how, where and when questions are asked[48]. The interpretation of 'good' absolute patient feedback scores should, therefore, not lead to complacency and the conclusion that improvements need not be considered.

The challenges of communicating risk are well known. Public understanding of risk is generally low and whilst reviews have shown that the way risk is presented affects risk perceptions[49,50], even immediately after being provided with CVD risk information one in four people still have an inaccurate perception of their risk[51] and one in ten change their perceived risk in the opposite direction to the feedback they receive[52]. The confusion around the risk scores seen in this study may therefore reflect a combination of how the risk is presented by healthcare professionals and how individuals interpret it within the context of

For peer review only - http://bmjopen.bmj.com/site/about/guidelines.xhtml

the NHS Health Check. The finding that knowing the CVD risk score was not sufficient to motivate behaviour change is also consistent with guidance from the National Institute for Health and Clinical Excellence (NICE) on behaviour change[53] and previous systematic reviews[54,14].

Implications for clinicians, policymakers and future research

Whilst participants were generally very supportive of the NHS Health Check programme and examples of behaviour change were reported, this study highlights a number of areas where improvements could be made. In particular, the finding that a number of patients were unaware of the programme or had misunderstood the extent and purpose of the NHS Health Check suggests that more proactive communications may be needed to raise awareness of the programme overall and that patients need additional clarity about the programme when being invited. The expectation that the NHS Health Check would be a more general health check also raises questions about whether the programme should be expanded to cover other areas of health. The lack of clarity around follow-up and reports that participants would have liked their healthcare professionals to be more pro-active in supporting them to make lifestyle changes additionally suggests that there are potential missed opportunities to support behaviour change. This may be in part due to a lack of appropriate services to refer patients to but may also reflect a need for additional training for those delivering the NHS Health Check. Finally, the confusion around the CVD risk score amongst patients highlights the potential limitations of relying on the risk score alone as a trigger for facilitating behaviour change within NHS Health Checks and the need for adequate training and time for healthcare professionals to help patients understand their risk in line with best practice guidance[50,55]. Further research is also needed to determine whether different communication strategies, such as heart age[56], improve understanding and subsequent behaviour change.

ACKNOWLEDGEMENTS

We thank our patient and public representatives Kathryn Lawrence and Chris Robertson for providing helpful comments on the findings and the NHS Health Checks Expert Scientific and Clinical Advisory Panel working group for providing us with the initial literature search conducted by Public Health England. We would also like to thank Anna Knack, Research Assistant at RAND Europe, for her excellent research support, and Emma Pitchforth for her helpful comments on our analysis.

For peer review only - http://bmjopen.bmj.com/site/about/guidelines.xhtml

Contributors

JUS developed the protocol, screened articles for inclusion, extracted and synthesised the quantitative and qualitative data, interpreted the findings and wrote the first draft of the manuscript. EH screened articles for inclusion, extracted and synthesised the qualitative data, interpreted the findings and critically revised the manuscript. CMa extracted and synthesised the qualitative data and critically revised the manuscript. AM screened articles for inclusion, interpreted the findings and critically revised the manuscript. CS, CM, FW, SG and JM developed the protocol, interpreted the findings and critically revised the manuscript.

Funding

This work was funded by a grant from Public Health England. JUS was funded by an NIHR Clinical Lectureship and FW by an NIHR Clinician Scientist award. The views expressed in this publication are those of the authors and not necessarily those of the NHS, the NIHR or the Department of Health.

All researchers were independent of the funding body and the funder had no role in data collection, analysis and interpretation of data; in the writing of the report; or decision to submit the article for publication.

Data sharing

All data are available from the reports or authors of the primary research. No additional data is available.

Competing Interests

All authors have completed the Unified Competing Interest form at *www.icmje.org/coi_disclosure.pdf* (available on request from the corresponding author) and declare that (1) they have no support from or relationships with companies that might have an interest in the submitted work in the previous 3 years; (2) their spouses, partners, or children have no financial relationships that may be relevant to the submitted work; and (3) they have no non-financial interests that may be relevant to the submitted work.

The corresponding author has the right to grant on behalf of all authors and does grant on behalf of all authors, an exclusive licence on a worldwide basis to the BMJ Publishing Group Ltd and its Licensees to permit this article (if accepted) to be published in BMJ editions and

For peer review only - http://bmjopen.bmj.com/site/about/guidelines.xhtml

any other BMJPGL products and sub-licences to exploit all subsidiary rights, as set out in our licence (http://resources.bmj.com/bmj/authors/checklists-forms/licence-for-publication).

All authors had full access to all of the data in the study and can take responsibility for the integrity of the data and the accuracy of the data analysis

The corresponding author affirms that the manuscript is an honest, accurate, and transparent account of the study being reported; that no important aspects of the study have been omitted; and that any discrepancies from the study as planned (and, if relevant, registered) have been explained.

FIGURE LEGENDS

Figure 1. PRISMA flow diagram

REFERENCES

1 Public Health England. NHS Health Check: Best Practice Guidance. Published Online First: 2017.http://www.healthcheck.nhs.uk/commissioners_and_providers/guidance/national_guidance1/

2 Hippisley-Cox J, Coupland C, Vinogradova Y, *et al.* Predicting cardiovascular risk in England and Wales: prospective derivation and validation of QRISK2. *BMJ* 2008;**336**:1475–82.

3 Department of Health. Economic Modelling for Vascular Checks. 2008.www.healthcheck.nhs.uk/document.php?o=225

4 Capewell S, McCartney M, Holland W. Invited debate: NHS Health Checks-a naked emperor? *J Public Health (Oxf)* 2015;**37**:187–92.

5 Dalton AR, Marshall T, McManus RJ. The NHS Health Check programme: a comparison against established standards for screening. *Br J Gen Pract* 2014;**64**:530–1.

6 Professor the Lord Darzi. High quality care for all: NHS Next Stage Review final report. 2008. http://webarchive.nationalarchives.gov.uk/20130107105354/http:/www.dh.gov.uk/en/Publicationsandstatistics/Publications/PublicationsPolicyAndGuidance/DH_085825

7 Institute of Medicine. Corssing the quality chasm: a new health system for the 21st

For peer review only - http://bmjopen.bmj.com/site/about/guidelines.xhtml

century. 2001. https://www.nationalacademies.org/hmd/~/media/Files/Report Files/2001/Crossing-the-Quality-Chasm/Quality Chasm 2001 report brief.pdf

8 Campbell SM, Roland MO, Buetow SA. Defining quality of care. *Soc Sci Med* 2000;**51**:1611–25.

9 Doyle C, Lennox L, Bell D. A systematic review of evidence on the links between patient experience and clinical safety and effectiveness. *BMJ Open* 2013;**3**:1–18.

10 Llanwarne NR, Abel GA, Elliott MN, *et al.* Relationship between clinical quality and patient experience: analysis of data from the english quality and outcomes framework and the National GP Patient Survey. *Ann Fam Med* 2013;**11**:467–72.

11 Critical Appraisal Skills Programme (CASP) Checklists. http://www.casp-uk.net/casp-tools-checklists

12 Cochrane Methods. Cochrane Supplemental Handbook Guidance. http://methods.cochrane.org/qi/supplemental-handbook-guidance (accessed 27 Apr2017).

13 Deeks JJ, Dinnes J, D'Amico R, *et al.* Evaluating non-randomised intervention studies. *Health Technol Assess* 2003;**7**:iii–x, 1-173.

14 Usher-Smith JA, Silarova B, Schuit E, *et al.* Impact of provision of cardiovascular disease risk estimates to healthcare professionals and patients: a systematic review. *BMJ Open* 2015;**5**:e008717.

15 Malpass A, Shaw A, Sharp D, *et al.* 'Medication career' or 'moral career'? The two sides of managing antidepressants: a meta-ethnography of patients' experience of antidepressants. *Soc Sci Med* 2009;**68**:154–68.

16 Thomas J, Harden A. Methods for the thematic synthesis of qualitative research in systematic reviews. *BMC Med Res Methodol* 2008;**8**:45.

17 Krska J, du Plessis R, Chellaswamy H. Views and experiences of the NHS Health Check provided by general medical practices: cross-sectional survey in high-risk patients. *J Public Health (Bangkok)* 2015;**37**:210–7.

18 Baker C, Loughren EA, Crone D, *et al.* Patients' perceptions of a NHS Health Check in the primary care setting. *Qual Prim Care* 2014;**22**:232–7.

19 Cowper. The NHS Health Check Leadership Forum : Summary and Findings. *NHS Heal Check Leadersh Forum* 2013.

20 Trivedy C, Vlaev I, Seymour R, *et al.* An evaluation of opportunistic health checks at cricket matches: the Boundaries for Life initiative. *Sport Soc* 2016;**437**:1–9.

21 NHS Greenwich. Evaluation of NHS Health Check PLUS Community Outreach

For peer review only - http://bmjopen.bmj.com/site/about/guidelines.xhtml

Programme in Greenwich. 2011;:1–61.

22 LGA - East Riding. Public health transformation twenty months on: adding value to tackle local health needs. Published Online First: 2015.http://www.local.gov.uk/documents/10180/6869714/L15_15+Public+health+transformation+twenty+months+on_WEB_39693.pdf/7bb8060e-9a7b-4b85-8099-e854be74cfb5

23 Health Diagnostics. A Picture Of Health NHS Health Checks Case Study : Case study Introduction : National inequalities in health check provision. 2014.

24 Taylor J, Krska J, MacKridge A. A community pharmacy-based cardiovascular screening service: Views of service users and the public. *Int J Pharm Pract* 2012;**20**:277–84.

25 Corlett SA, Krska J. Evaluation of NHS Health Checks provided by community pharmacies. *J Public Health (Bangkok)* 2015;:1–8.

26 Ismail H, Atkin K. The NHS Health Check programme: Insights from a qualitative study of patients. *Heal Expect* 2015;**19**:345–55.

27 Jenkinson CE, Asprey A, Clark CE, *et al.* Patients' willingness to attend the NHS cardiovascular health checks in primary care: a qualitative interview study. *BMC Fam Pract* 2015;**16**:33.

28 McNaughton RJ, Oswald NT, Shucksmith JS, *et al.* Making a success of providing NHS Health Checks in community pharmacies across the Tees Valley: a qualitative study. *BMC Health Serv Res* 2011;**11**:1–7.

29 Perry C, Thurston M, Alford S, *et al.* The NHS health check programme in England: a qualitative study. *Health Promot Int* 2014;**31**:106–15.

30 Riley R, Coghill N, Montgomery A, *et al.* Experiences of patients and healthcare professionals of NHS cardiovascular health checks: A qualitative study. *J Public Health (Bangkok)* 2015;**38**:543–51.

31 Riley R, Coghill N, Montgomery A, *et al.* The provision of NHS health checks in a community setting: an ethnographic account. *BMC Health Serv Res* 2015;**15**:1–10.

32 Shaw RL, Pattison HM, Holland C, *et al.* Be SMART: examining the experience of implementing the NHS Health Check in UK primary care. *BMC Fam Pract* 2015;**16**:1.

33 Alford S, Catherine P. Knowsley at Heart community NHS health checks : Behaviour change evaluation. 2010.http://chesterrep.openrepository.com/cdr/handle/10034/118071

34 Chipchase L, Hill P, Waterall J. An insight into the NHS Health Check Programme in

For peer review only - http://bmjopen.bmj.com/site/about/guidelines.xhtml

Birmingham; Summar report. 2011.

35 Oswald N, Mcnaughton R, Watson P, *et al.* Tees Vascular Assessment Programme Evaluation. 2010.

36 Strutt E. *Patient-centred care: patients' experiences of and responses to the National Health Service (NHS) Health Check programme in general practice.* 2011.http://etheses.dur.ac.uk/3246

37 McNaughton RJ, Shucksmith J. Reasons for (non)compliance with intervention following identification of 'high-risk' status in the NHS Health Check programme. *J Public Heal (United Kingdom)* 2015;**37**:218–25.

38 Britten N, Campbell R, Pope C, *et al.* Using meta ethnography to synthesise qualitative research: a worked example. *J Health Serv Res Policy* 2002;**7**:209–15.

39 Groves RM, Peytcheva E. The impact of nonresponse rates on nonresponse bias: A meta-analysis. *Public Opin Q* 2008;**72**:167–89.

40 Davern M. Nonresponse rates are a problematic indicator of nonresponse bias in survey research. *Health Serv Res* 2013;**48**:905–12.

41 Cleary P. Satisfaction may not suffice!: a commentary on a patient's perspective. *Int J Tech Assess Heal Care* 1998;**14**:35–7.

42 Salisbury C, Wallace M, Montgomery AA. Patients' experience and satisfaction in primary care: secondary analysis using multilevel modelling. *BMJ* 2010;**341**:c5004–c5004.

43 Sitzia J, Wood N. Patient satisfaction: a review of issues and concepts. *Soc Sci Med* 1997;**45**:1829–43.

44 Althubaiti A. Information bias in health research: definition, pitfalls, and adjustment methods. *J Multidiscip Healthc* 2016;**9**:211–7.

45 Roland M, Campbell J, Burt J. Improving patient experience in primary care: a multi-method programme of research on the measurement and improvement of patient experience. *NIHR PGfAR*;:In press.

46 Dougall A, Russell A, Rubin G, *et al.* Rethinking patient satisfaction: patient experiences of an open access flexible sigmoidoscopy service. *Soc Sci Med* 2000;**50**:53–62.

47 Burt J, Newbould J, Abel G, *et al.* Investigating the meaning of 'good' or 'very good' patient evaluations of care in English general practice: a mixed methods study. *BMJ Open* 2017;:In press.

48 Schneider H, Palmer N. Getting to the truth? Researching user views of primary health

For peer review only

For peer review only - http://bmjopen.bmj.com/site/about/guidelines.xhtml

care. *Health Policy Plan* 2002;**17**:32–41.

49 Waldron C-A, van der Weijden T, Ludt S, *et al.* What are effective strategies to communicate cardiovascular risk information to patients? A systematic review. *Patient Educ Couns* 2011;**82**:169–81.

50 Zipkin DA, Umscheid CA, Keating NL, *et al.* Evidence-based risk communication: a systematic review. *Ann Intern Med* 2014;**161**:270–80.

51 Hussein HM, Harris-Lane P, Abdelmoula MM, *et al.* Accuracy of self-perception of cardiovascular risk in the community. *J Vasc Interv Neurol* 2008;**1**:106–12.

52 Avis NE, Smith KW, McKinlay JB. Accuracy of perceptions of heart attack risk: what influences perceptions and can they be changed? *Am J Public Health* 1989;**79**:1608–12.

53 National Institute for Health and Care Excellence. Public Health Guideline - Behaviour change: individual approaches. 2014. www.nice.org.uk/guidance/ph49

54 Willis A, Davies M, Yates T, *et al.* Primary prevention of cardiovascular disease using validated risk scores: a systematic review. *J R Soc Med* 2012;**105**:348–56.

55 Fagerlin A, Zikmund-Fisher BJ, Ubel PA. Helping patients decide: Ten steps to better risk communication. *J Natl Cancer Inst* 2011;**103**:1436–43.

56 Wells S, Kerr A, Eadie S, *et al.* 'Your Heart Forecast': a new approach for describing and communicating cardiovascular risk? *Heart* 2010;**96**:708–13.

For peer review only

For peer review only - http://bmjopen.bmj.com/site/about/guidelines.xhtml

Table 1. Features of studies reporting results of participant surveys

Study/ year	Type of article	Setting	*n*	Recruitment	Response rate (%)	Overall quality
Baker 2014[18]	Journal article	83 general practices	1,011	Survey sent to all patients who had completed an NHS Health Check within a 2-month period	43	High
Corlett 2015[25]	Journal article	Pharmacy-based NHS Health Checks	66	Survey sent to all those who had attended an NHS Health Check within a 4 week period	35	High
Cowper 2013[19]	Case study	NHS Health Checks in County Durham	483	No details provided	Not given	Low
Krska 2015[17]	Journal article	16 general practices in North West England	434	All patients with estimated 10-year CVD risk > 20% from the 16 practices were sent a postal survey regardless of whether they had attended an NHS Health Check or not	23.4	High
LGA – East Riding[22]	Case study	Outreach NHS Health Check clinics at leisure centres, community centres and workplace settings	Not given	No details provided	Not given	Low
NHS Greenwich[21]	Evaluation report	Outreach clinics	540	Questionnaire distributed at community NHS Health Check venues	Not given	Medium
NHS Greenwich[21]	Evaluation report	Outreach clinics	72	Questionnaire distributed at community NHS Health Check venues	Not given	Medium
'A picture of Health'[23]	Case study	General practice-based pilot of point-of-care NHS Health Checks in Tyne and Wear	281	No details provided	Not given	Low
Taylor 2012[24]	Journal article	Pharmacy-based NHS Health Checks	97	Pharmacists gave invitation packs to all those who attended an NHS Health Check during the first six months	37.4	High
Trivedy 2016[20]	Journal article	Outreach NHS Health Check clinics at cricket grounds	513	Participants were asked to complete an anonymous questionnaire immediately after their NHS Health Check	Not given	Medium

For peer review only

For peer review only - http://bmjopen.bmj.com/site/about/guidelines.xhtml

Table 2. Findings from studies reporting results of participant surveys

Domain	Result
Overall experience / satisfaction	91.7% rated the overall experience highly[18]<br>Almost all viewed their experience positively[25]<br>92% rated experience as good or very good[22]<br>Almost all reported a positive experience[24]<br>83% rated their experience as excellent[20]<br>82.2% were very satisfied[19]<br>97% satisfied or very satisfied overall[21]<br>95% satisfied or very satisfied[21]<br>'High levels' of satisfaction[23]
Recommend to others	99.6% would recommend to others[19]<br>78% likely to recommend to others[23]<br>100% would recommend to others[20]
Benefit	99% felt they had benefited[24]<br>85.6% felt they had benefited[17]<br>90.2% felt the NHS Health Check was worth attending[18]<br>90% likely or very likely to return if invited back[21]
Time / opportunity to ask questions	88.0% agreed they had the time to ask questions[18]<br>92% felt they were given enough time[25]<br>94% were able to ask all their questions[25]<br>99.7% felt they were given enough time[24]<br>89.6% felt they were given enough time[17]<br>90.2% were able to ask all their questions[17]<br>9% had unanswered questions[25]<br>10.8% had unanswered questions[24]<br>14.7% still had questions about their risk of heart disease[17]<br>7.4% had concerns that had not been dealt with[17]
Understanding and recall of CVD risk	97% understood everything[25]<br>59% could recall their actual CVD score[25]<br>91.9% understood everything discussed[17]<br>83% felt the Health Check had helped them to understand their risk of heart disease[17]<br>61.9% rated their understanding of the CVD risk score highly[18]
Location and timing of appointments	69.5% rated the location of Doctor's surgery highly[18]<br>70.7% rated the time and availability of appointments highly[18]<br>93.8% agreed that screening had been done in a suitable place[24]<br>86% felt the location gave enough privacy[20]
Staff	93.8% rated confidence in staff knowledge[18]<br>92% reported that staff were helpful, friendly and clear about the service during their Health Check[22]<br>100% felt they were treated with dignity[20]<br>99% felt comfortable discussing their lifestyle[24]<br>93.6% felt comfortable discussing their lifestyle[17]<br>13.5% would have liked more support changing lifestyle[17]

For peer review only

Table 3. Features of qualitative studies describing patient experiences of NHS Health Checks

Author, year	Type of report	Location of study	Setting of NHS Health Check	Data collection method	Setting for data collection	*n*	Method of recruitment to study	Participant characteristics	Method of analysis	Overall quality
Alford 2010[33]	Evaluation report	Knowsley	Community	Interviews and focus groups	Not given	36	No details given	19 female, 17 male<br>13 high risk score, 23 low risk score	Thematic analysis	Medium
Baker 2014[18]	Journal article	Gloucester	83 general practices	Content analysis of cross-sectional survey	N/A	1,011 (43%)	Survey sent to all patients who had completed an NHS Health Check within a 2 month period	55.2% female<br>19% 56-60 years<br>10.8% 40-45 years<br>96% white British	Thematic analysis	High
Chipchase 2011[34]	Report	East and North Birmingham	2 general practices	Face-to-face semi-structured interviews	GP surgery	10	Attendees to NHS Health Checks in the first two weeks of February 2011 received a recruitment letter	8 female, 2 male	Interpretative phenomeno-logical analysis	High
Corlett 2015[25]	Journal article	London	4 pharmacies	Telephone interviews with sample of survey respondents	On the telephone	19	Invitation for a semi-structured telephone interview included with survey sent to all those who had attended an NHS Health Check within a 4 week period	Not given	Thematic analysis using framework approach	Medium
Greenwich 2011[21]	Report	Greenwich	Community	Open ended questionnaire, focus groups and in-depth phone interviews	Not given	612 survey responses<br>4 focus groups and 31 interviews	Recruited from community outreach services providing NHS Health Checks	Ethnic minority participants: 42% female	Based on Health Belief Model	Medium
Ismail and Atkin 2015[26]	Journal article	Not specified	General practices	Semi-structured interviews	Participants' homes or NHS premises	45 baseline<br>38 follow-up	Purposive sampling from a list provided by 5 participating general practices	21 female, 24 male.<br>Average age: 58.<br>Ethnicity: 37 White, 5 South Asian and 3 African Caribbean	Framework analysis	High
Jenkinson 2015[27]	Journal article	Torbay	4 general practices	Telephone or face-to-face interviews	On the telephone or participants' homes	17	Letters of invitation sent to a random sample identified by general practices from lists stratified by age and gender of those who had not responded to an invitation to an NHS	12 females, 5 males<br>6 employed, 1 unemployed, 10 retired	Thematic analysis	High

For peer review only

For peer review only - http://bmjopen.bmj.com/site/about/guidelines.xhtml

Author, year	Type of report	Location of study	Setting of NHS Health Check	Data collection method	Setting for data collection	*n*	Method of recruitment to study	Participant characteristics	Method of analysis	Overall quality
							Health Check within 4 weeks.			
Krska 2015[17]	Journal article	Sefton, an area of North West England	16 general practices	Postal survey with free text responses	N/A	434 (23.4%)	All patients with estimated 10 year CVD risk > 20% from the 16 practices were sent a postal survey regardless of whether they had attended an NHS Health Check or not	19% female<br>68.2% over 65<br>99.5% white<br>7.7% highest quintile of deprivation<br>13.7% lowest quintile	Categorisation of responses	Medium
McNaughton 2015[37]	Journal article	North East of England (non-specific location)	5 general practices	Semi-structured interviews	Not given	29	Invitations to patients from five general practices who had received an NHS Health Check and had an estimated 10 year CVD risk >20%	10 females, 19 males<br>24 over 65 years<br>13 in least deprived quintile	Thematic analysis	High
Oswald 2010[35]	Evaluation report	Teesside	General practices or pharmacies	Telephone semi-structured interviews	On the telephone	8	Invited by general practices or pharmacies or from a list of patients who had attended an NHS Health Check and agreed to take part in the service evaluation	6 had attended general practices and 2 pharmacies	Thematic analysis	Medium
Perry 2014[29]	Journal article	Knowsley	Community	Interviews and focus groups	Not given	36	Letter or telephone invitation to all 38 people who were at high risk of CVD and had attended an NHS Health Check in the past 12-18 months were invited. The remaining attendees at low risk of CVD were purposively sampled for gender, age , risk score.	3 focus groups: 1 for high risk scores (6 males), 2 for low risk scores (17 females and 7 males)<br>6 semi-structured interviews (2 females and 4 males with high risk score)	Thematic analysis	High
Riley 2015[31]	Journal article	Bristol inner-city	Community	Semi-structured interviews	Community venues or participants' homes	16	Participants were recruited via their attendance of community outreach events.	7 females, 9 males<br>All from black and minority ethnic populations	Thematic analysis	High
Riley 2015[30]	Journal article	Bristol	General practices	Face-to-face and telephone	On the telephone or	28	Purposive sampling from those identified through a	16 females, 12 males<br>23 White British	Thematic analysis	High

For peer review only - http://bmjopen.bmj.com/site/about/guidelines.xhtml

Author, year	Type of report	Location of study	Setting of NHS Health Check	Data collection method	Setting for data collection	*n*	Method of recruitment to study	Participant characteristics	Method of analysis	Overall quality
				semi-structured interviews	in participants' homes		search of patient records for patients who had undertaken an NHS Health Check within the previous 6 months	11 most deprived quintile<br>11 high (>20%) CVD risk		
Shaw 2015[32]	Journal article	Birmingham and Black Country	General practices and community	Semi-structured interviews	Not given	23	Patients who had attended an NHS Health Check were invited by practice managers or lead clinicians	High black and minority ethnic population and high levels of deprivation	Thematic analysis	High
Strutt 2011[36]	Masters thesis	Darlington, Co. Durham, UK	2 general practices	Semi-structured face-to-face interviews	Participants' homes	16	Invitation letters or telephone	7 females, 9 males<br>White, South-Asian, and Middle Eastern	Thematic analysis	High

For peer review only

For peer review only - http://bmjopen.bmj.com/site/about/guidelines.xhtml

Table 4. Studies contributing to each of the qualitative themes

	Unmet expectations	Limited understanding of the risk score	Preference for better information	Potential trigger for behaviour change	Confusion around follow-up
Alford 2010[33]			●	●	●
Baker 2014[18]	●	●	●	●	
Chipchase 2011[34]	●	●	●	●	
Corlett 2015[25]		●	●	●	
Greenwich 2011[21]			●	●	●
Ismail and Atkin 2015[26]	●	●		●	●
Jenkinson 2015[27]		●			
Krska 2015[17]	●	●			
McNaughton 2015[37]			●	●	
Oswald 2010[35]	●				●
Perry 2014[29]		●	●	●	
Riley 2015[31]		● (a)	●	●	
Riley 2015[30]	● (b)				
Shaw 2015[32]		●	●	●	●
Strutt 2011[36]	●	●	●	●	

For peer review only

Box 1. Reasons provided by participants for not making lifestyle changes

- Older participants feeling that making changes to their lifestyle was unnecessary [37]
- Healthy eating information was too generic [37]
- Guidance they had been given was likely to be subject to change [37]
- Co-morbidities which made physical activity difficult [37]
- Psychosocial circumstances, e.g. bereavement, stress or socio-economic barriers, such as shift work or unemployment [29,30]
- Having previously been offered a behaviour intervention strategy [26]
- Cost of eating fresh fruit and vegetables [26]
- Difficulty incorporating changes into their daily lives [29]
- Underlying medical conditions [29]

For peer review only - http://bmjopen.bmj.com/site/about/guidelines.xhtml

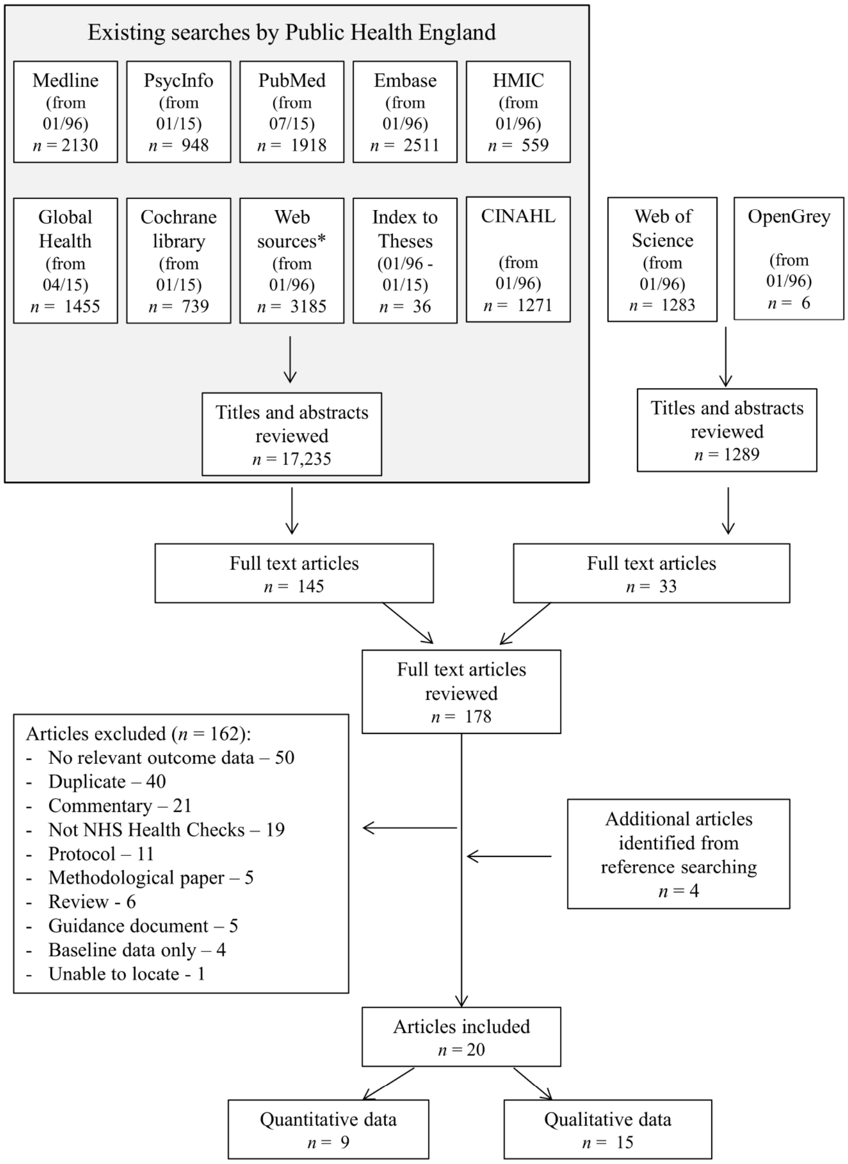

Figure 1. PRISMA diagram

231x308mm (300 x 300 DPI)

Appendix 1 – Search strategies

Database	Search strategy
Ovid Medline	1. health check*.tw.<br>2. (diabetes adj3 screen*).tw.<br>3. (cardiovascular adj3 screen*).tw.<br>4. (population adj2 screen*).tw.<br>5. (risk factor adj3 screen*).tw.<br>6. (opportunistic adj3 screen*).tw.<br>7. medical check*.tw.<br>8. general check*.tw.<br>9. periodic health exam*.tw.<br>10. annual exam*.tw.<br>11. annual review*.tw.<br>12. NHSHC.tw.<br>13. 1 or 2 or 3 or 4 or 5 or 6 or 7 or 8 or 9 or 10 or 11 or 12<br>14. cardiovascular adj3 prevention.tw.<br>15. (primary care or general practice or primary healthcare).tw<br>16. 14 and 15<br>17. Cardiovascular Diseases/ AND Primary Prevention/<br>18. 16 or 17<br>19. 13 or 18
PubMed	1. health check*<br>2. diabetes screen*<br>3. cardiovascular screen*<br>4. population screen*<br>5. risk factor screen*<br>6. opportunistic screen*<br>7. medical check*<br>8. general check*<br>9. periodic health exam*<br>10. annual exam*<br>11. annual review*<br>12. NHSHC<br>13. 1 or 2 or 3 or 4 or 5 or 6 or 7 or 8 or 9 or 10 or 11 or 12<br>14. Cardiovascular Diseases AND Primary Prevention[MeSH Terms]<br>15. "primary care"[Text Word] OR "general practice"[Text Word] OR "primary healthcare"[Text Word])<br>16. (cardiovascular[Text Word] AND prevention[Text Word])<br>17. #15 and #16<br>18. #14 or #17<br>19. #13 or #18
Ovid Embase	1. health check*.tw.<br>2. (diabetes adj3 screen*).tw.<br>3. (cardiovascular adj3 screen*).tw.<br>4. (population adj2 screen*).tw.

For peer review only

	5. (risk factor adj3 screen*).tw.<br>6. (opportunistic adj3 screen*).tw.<br>7. medical check*.tw.<br>8. general check*.tw.<br>9. periodic health exam*.tw.<br>10. annual exam*.tw.<br>11. annual review*.tw.<br>12. NHSHC.tw.<br>13. periodic medical examination/<br>14. 1 or 2 or 3 or 4 or 5 or 6 or 7 or 8 or 9 or 10 or 11 or 12 or 13<br>15. cardiovascular adj3 prevention.tw.<br>16. (primary care or general practice or primary healthcare).tw<br>17. 15 and 16<br>18. cardiovascular disease/ AND primary prevention/<br>19. 17 or 18<br>20. 14 or 19
Ovid HMIC	1 "health check*".af.<br>2 health checks/<br>3 (cardiovascular or vascular or heart or diabetes or stroke).af.<br>4 (screen* or risk).af.<br>5 3 AND 4<br>6 1 OR 2 or 5<br>7 cardiovascular adj3 prevention.tw.<br>8 (primary care or general practice or primary healthcare).tw<br>9 7 and 8<br>10 Cardiovascular diseases/ AND exp preventive medicine/<br>11 9 or 10<br>12 6 or 11
EBSCO CINAHL	S10 S1 OR S2 OR S9<br>S9 S5 OR S8<br>S8 S6 AND S7<br>S7 (MH "Preventive Health Care+")<br>S6 (MH "Cardiovascular Diseases+")<br>S5 S3 AND S4<br>S4 "primary care" or "general practice" or "primary healthcare"<br>S3 TX cardiovascular N3 prevention<br>S2 (diabetes N3 screen*) OR (cardiovascular N3 screen*) OR (population N2 screen*) OR (risk factor N3 screen*) OR (opportunistic N3 screen*) OR “medical check*” OR “general check*” OR “periodic health exam*” OR “annual exam*” OR "annual review*" OR NHSHC<br>S1 health check*
EBSCO Global Health	S10 S6 OR S19 OR S3 Limiters - Publication Year: 2016<br>S9 S7 AND S8<br>S8 DE "preventive medicine"<br>S7 DE "cardiovascular diseases"<br>S6 S4 AND S5<br>S5 "primary care" or "general practice" or "primary healthcare"

For peer review only

	S4 TX cardiovascular N3 prevention<br>S3 S1 OR S2<br>S2 (diabetes N3 screen*) OR (cardiovascular N3 screen*) OR (population N2 screen*) OR (risk factor N3 screen*) OR (opportunistic N3 screen*) OR “medical check*” OR “general check*” OR “periodic health exam*” OR “annual exam*” OR "annual review*" OR NHSHC<br>S1 health check*
HDAS PsycInfo	1 "health check*".af.<br>2 PHYSICAL EXAMINATION/<br>3 HEALTH SCREENING/<br>4 "diabetes screen*".af<br>5 "cardiovascular screen*".af<br>6 "population screen*".af<br>7 ("opportunistic* screen*" OR "risk factor screen*").af<br>8 ("medical check*" OR "general check*" OR "periodic health exam*" OR "annual exam*" OR "annual review*" OR NHSHC).af<br>9 1 OR 2 OR 3 OR 4 OR 5 OR 6 OR 7 OR 8<br>10 cardiovascular.ti,ab<br>11 prevention.ti,ab<br>12 10 AND 11<br>13 CARDIOVASCULAR DISORDERS/<br>14 PREVENTIVE MEDICINE/<br>15 13 AND 14<br>16 12 OR 15<br>17 9 OR 16
Web of Science, Science Citation Index	“health check*” OR “diabetes screen*” OR “cardiovascular screen*” OR “population screen*” OR “risk factor screen*” OR “Opportunistic screen*” OR “medical check*” OR “general check*” OR “periodic health exam*” OR “annual exam*” OR “annual review*” OR NHSHC<br>OR<br>(Cardiovascular NEAR/3 prevention) AND (“primary care” OR “general practice” OR “primary healthcare”)<br>Limit to: England, Scotland, Wales, North Ireland
Cochrane Library (Wiley)	#1 "health check*"<br>#2 (diabetes next/3 screen*) or (cardiovascular next/3 screen*) or (population next/2 screen*) or (opportunistic next/2 screen*) or ("risk factor" next/3 screen*) or "medical check*" or "general check*" or "periodic health exam*" or "annual exam*" or "annual review*" or NHSHC<br>#3 cardiovascular adj3 prevention.tw.<br>#4 (primary care or general practice or primary healthcare).tw<br>#5 #3 and #4<br>#6 MeSH descriptor: [Cardiovascular Diseases] this term only<br>#7 MeSH descriptor: [Primary Prevention] explode all trees<br>#8 #6 and #7<br>#9 #5 or #8<br>#10 #1 or #2 or #9

For peer review only

NHS Evidence	“health check*” OR cardiovascular prevention primary care
TRIP database	“health check*” OR cardiovascular prevention primary care
Google Scholar	"nhs health check"<br>cardiovascular “health check”<br>cardiovascular prevention “primary care”
Google	"nhs health check"<br>cardiovascular prevention “primary care”<br>cardiovascular “health check”
Clinical trials.gov and ISRCDN registry	“health check”

For peer review only

Appendix 2 – Quality assessment of quantitative studies

Author, date	Study addressed a clearly focused issue	Use of an appropriate method	Recruitment	Exposure measurement	Outcome measurement	Confounding factors	Applicability to England	Overall
Baker 2014	High	Medium	High	Medium	Medium	Low	Medium	High
Corlett 2015	High	High	High	High	High	Low	Low	High
Cowper 2013	High	Low	Low	Low	Low	Low	Medium	Low
Krska 2015	High	High	High	Medium	High	Low	Medium	High
LGA – East Riding 2015	High	Low	Low	Low	Low	Low	Low	Low
NHS Greenwich	Medium	Medium	Medium	Medium	Medium	Low	Low	Medium
‘A picture of Health’	Medium	Low	Low	Low	Low	Low	Low	Low
Taylor 2012	High	High	Medium	High	High	Low	Medium	High
Trivedy 2016	Medium	Medium	Medium	High	Medium	Low	Low	Medium

• Low ● Medium ⬤ High

For peer review only

Appendix 3 – Quality assessment of qualitative studies

Author and date	Study addressed a clearly focused issue	Appropriateness of qualitative method	Design	Recruitment	Consideration of relationship between research and participants	Ethical issues	Rigor of data analysis	Clarity of statement of findings	Overall
Alford 2010	⬤	⬤	⬤	•	•	•	•	●	Medium
Baker 2014	⬤	⬤	⬤	⬤	n/a	•	⬤	⬤	High
Chipchase 2011	⬤	⬤	⬤	●	●	⬤	⬤	●	High
Corlett 2015	⬤	⬤	⬤	●	•	●	●	●	Medium
Greenwich 2011	⬤	⬤	⬤	●	•	•	•	●	Medium
Ismail and Atkin 2015	⬤	⬤	⬤	⬤	●	⬤	⬤	⬤	High
Jenkinson 2015	⬤	⬤	⬤	⬤	•	⬤	⬤	⬤	High
Krska 2015	⬤	⬤	●	⬤	n/a	●	●	●	Medium
McNaughton 2015	⬤	⬤	⬤	⬤	•	⬤	⬤	⬤	High
Oswald 2010	⬤	⬤	⬤	●	•	●	●	●	Medium
Perry 2014	⬤	⬤	⬤	⬤	•	•	⬤	⬤	High
Riley 2015 (JPH)	⬤	⬤	⬤	●	●	⬤	⬤	⬤	High
Riley 2015 (BMC HSR)	⬤	⬤	⬤	●	●	⬤	⬤	⬤	High
Shaw 2015	⬤	⬤	⬤	●	•	⬤	⬤	⬤	High
Strutt 2011	⬤	⬤	⬤	⬤	⬤	⬤	⬤	⬤	High

• Low ● Medium ⬤ High

PRISMA 2009 Checklist

Section/topic	#	Checklist item	Reported on page #
**TITLE**			
Title	1	Identify the report as a systematic review, meta-analysis, or both.	1
**ABSTRACT**			
Structured summary	2	Provide a structured summary including, as applicable: background; objectives; data sources; study eligibility criteria, participants, and interventions; study appraisal and synthesis methods; results; limitations; conclusions and implications of key findings; systematic review registration number.	2
**INTRODUCTION**			
Rationale	3	Describe the rationale for the review in the context of what is already known.	4
Objectives	4	Provide an explicit statement of questions being addressed with reference to participants, interventions, comparisons, outcomes, and study design (PICOS).	4
**METHODS**			
Protocol and registration	5	Indicate if a review protocol exists, if and where it can be accessed (e.g., Web address), and, if available, provide registration information including registration number.	5
Eligibility criteria	6	Specify study characteristics (e.g., PICOS, length of follow-up) and report characteristics (e.g., years considered, language, publication status) used as criteria for eligibility, giving rationale.	5
Information sources	7	Describe all information sources (e.g., databases with dates of coverage, contact with study authors to identify additional studies) in the search and date last searched.	5
Search	8	Present full electronic search strategy for at least one database, including any limits used, such that it could be repeated.	Appendix 1
Study selection	9	State the process for selecting studies (i.e., screening, eligibility, included in systematic review, and, if applicable, included in the meta-analysis).	5
Data collection process	10	Describe method of data extraction from reports (e.g., piloted forms, independently, in duplicate) and any processes for obtaining and confirming data from investigators.	5/6
Data items	11	List and define all variables for which data were sought (e.g., PICOS, funding sources) and any assumptions and simplifications made.	Table 1 and 2
Risk of bias in individual studies	12	Describe methods used for assessing risk of bias of individual studies (including specification of whether this was done at the study or outcome level), and how this information is to be used in any data synthesis.	6
Summary measures	13	State the principal summary measures (e.g., risk ratio, difference in means).	6
Synthesis of results	14	Describe the methods of handling data and combining results of studies, if done, including measures of consistency (e.g., I^2) for each meta-analysis.	6

For peer review only - http://bmjopen.bmj.com/site/about/guidelines.xhtml

PRISMA 2009 Checklist

Section/topic	#	Checklist item	Reported on page #
Risk of bias across studies	15	Specify any assessment of risk of bias that may affect the cumulative evidence (e.g., publication bias, selective reporting within studies).	5
Additional analyses	16	Describe methods of additional analyses (e.g., sensitivity or subgroup analyses, meta-regression), if done, indicating which were pre-specified.	N/A
**RESULTS**			
Study selection	17	Give numbers of studies screened, assessed for eligibility, and included in the review, with reasons for exclusions at each stage, ideally with a flow diagram.	6 and Fig 1
Study characteristics	18	For each study, present characteristics for which data were extracted (e.g., study size, PICOS, follow-up period) and provide the citations.	Table 1 and 2
Risk of bias within studies	19	Present data on risk of bias of each study and, if available, any outcome level assessment (see item 12).	Table 1 and 2 and Appendix 2
Results of individual studies	20	For all outcomes considered (benefits or harms), present, for each study: (a) simple summary data for each intervention group (b) effect estimates and confidence intervals, ideally with a forest plot.	N/A
Synthesis of results	21	Present results of each meta-analysis done, including confidence intervals and measures of consistency.	N/A
Risk of bias across studies	22	Present results of any assessment of risk of bias across studies (see Item 15).	N/A
Additional analysis	23	Give results of additional analyses, if done (e.g., sensitivity or subgroup analyses, meta-regression [see Item 16]).	N/A
**DISCUSSION**			
Summary of evidence	24	Summarize the main findings including the strength of evidence for each main outcome; consider their relevance to key groups (e.g., healthcare providers, users, and policy makers).	11
Limitations	25	Discuss limitations at study and outcome level (e.g., risk of bias), and at review-level (e.g., incomplete retrieval of identified research, reporting bias).	12
Conclusions	26	Provide a general interpretation of the results in the context of other evidence, and implications for future research.	13/14
**FUNDING**			
Funding	27	Describe sources of funding for the systematic review and other support (e.g., supply of data); role of funders for the systematic review.	14/15

For peer review only

For peer review only - http://bmjopen.bmj.com/site/about/guidelines.xhtml

PRISMA 2009 Checklist

From: Moher D, Liberati A, Tetzlaff J, Altman DG, The PRISMA Group (2009). Preferred Reporting Items for Systematic Reviews and Meta-Analyses: The PRISMA Statement. PLoS Med 6(6): e1000097. doi:10.1371/journal.pmed1000097

For more information, visit: **www.prisma-statement.org**.

For peer review only

For peer review only - http://bmjopen.bmj.com/site/about/guidelines.xhtml
